# A whole-genome sequence study identifies genetic risk factors for neuromyelitis optica

**DOI:** 10.1038/s41467-018-04332-3

**Published:** 2018-05-16

**Authors:** Karol Estrada, Christopher W. Whelan, Fengmei Zhao, Paola Bronson, Robert E. Handsaker, Chao Sun, John P. Carulli, Tim Harris, Richard M. Ransohoff, Steven A. McCarroll, Aaron G. Day-Williams, Benjamin M. Greenberg, Daniel G. MacArthur

**Affiliations:** 1 Translational Genome Sciences, Biogen, Cambridge, MA 02142 USA; 2 Program in Medical and Population Genetics, Broad Institute of Harvard and MIT, Cambridge, MA 02142 USA; 3 Stanley Center for Psychiatric Research, Broad Institute of MIT and Harvard, Cambridge, MA 02142 USA; 4 Department of Genetics, Harvard Medical School, Boston, MA 02115 USA; 5 Analytic and Translational Genetics Unit, Massachusetts General Hospital, Boston, MA 02114 USA; 6 Neuroimmunology, Acute Neurology and Pain, Biogen, Cambridge, MA 02142 USA; 7 Department of Neurology and Neurotherapeutics, Department of Pediatrics, University of Texas Southwestern Medical Center, Dallas, TX 75390 USA; 8 Present Address: Bioverativ, Waltham, MA 02451 USA; 9 Present Address: Third Rock Ventures, Boston, MA 02116 USA; 10 Present Address: Genetics and Pharmacogenomics, Merck, Boston, MA 02115 USA

## Abstract

Neuromyelitis optica (NMO) is a rare autoimmune disease that affects the optic nerve and spinal cord. Most NMO patients ( > 70%) are seropositive for circulating autoantibodies against aquaporin 4 (NMO-IgG+). Here, we meta-analyze whole-genome sequences from 86 NMO cases and 460 controls with genome-wide SNP array from 129 NMO cases and 784 controls to test for association with SNPs and copy number variation (total *N* = 215 NMO cases, 1244 controls). We identify two independent signals in the major histocompatibility complex (MHC) region associated with NMO-IgG+, one of which may be explained by structural variation in the complement component 4 genes. Mendelian Randomization analysis reveals a significant causal effect of known systemic lupus erythematosus (SLE), but not multiple sclerosis (MS), risk variants in NMO-IgG+. Our results suggest that genetic variants in the MHC region contribute to the etiology of NMO-IgG+ and that NMO-IgG+ is genetically more similar to SLE than MS.

## Abstract (web summary)

Neuromyelitis optica (NMO) is a rare autoimmune condition characterized by inflammation and demyelination of the optic nerve and the spinal cord. Here, Estrada et al. identify NMO susceptibility variants in the MHC region and find that autoantibody-positive NMO genetically overlaps with lupus.

## Introduction

Neuromyelitis optica (NMO) is a central nervous system autoimmune disorder that affects the optic nerves and spinal cord^1^. Once thought to be a clinical sub-class of multiple sclerosis (MS), NMO has now been recognized as a distinct clinical entity owing to the presence of antibody biomarkers (termed NMO-IgG) found in NMO but not MS cases^2^. Pathogenic water channel aquaporin 4 (AQP4) antibodies were identified within NMO-IgG, and sensitive cell-based assays demonstrated reactivity in > 70% of those with NMO syndrome manifestations. The estimated prevalence is 0.3–4.4 per 100,000, equivalent to 200,000–3 million cases worldwide^3^. NMO occurs preferentially in women ( > 80%) and appears most often in individuals between the ages of 30 and 40^4^. Less than 3% of NMO cases have one affected relative^3^. Heritability estimates have not been reported, but the small sibling recurrence rate indicates that NMO behaves more like a complex disease than a monogenic disease.

**Fig. 1:**
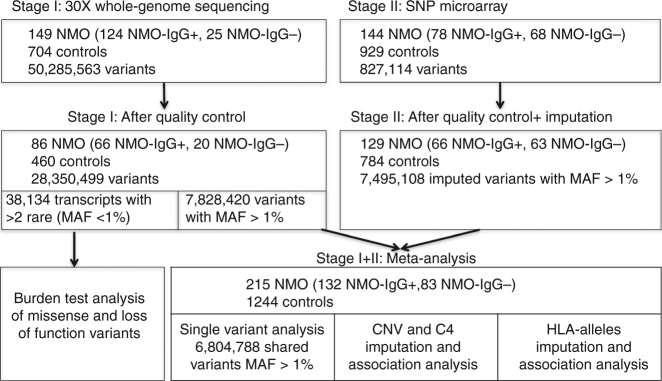
Description of samples, technology and methods used for the analysis of the two stages of our study. Abbreviations: NMO, neuromyelitis optica; NMO-IgG+, neuromyelitis optica positive for autoantibodies for AQP4; NMO-IgG−, neuromyelitis optica negative for autoantibodies for AQP4; MAF, minor allele frequency; SNP, single-nucleotide polymorphism. NMO cases were obtained from the Accelerated Cure Project. Stage I controls included neurological normal individuals from the Genomic Psychiatry Cohort. Stage II controls were neurological normal individuals from Coriell collections (Methods)

Just one genome-wide association study (GWAS) has been published for NMO (Korean cases)^5^, however, results were not significant, potentially because of the limited power and different ethnic background. To elucidate genetic factors driving NMO risk and to clarify the genetic architecture of this disease, we analyzed up to 6.8 million single-nucleotide polymorphisms (SNPs) and performed copy number variation (CNV) analysis on 215 cases and 1244 controls of European ancestry (Fig. 1). These 215 NMO cases may represent up to 23% of the total number of NMO cases in the United States. In this study, we find two independent significant genetic signals in the major histocompatibility complex (MHC) region associated with NMO. One of these signals may be explained by a common copy number event of the *C4A*/*C4B* genes. We also provide initial evidence that suggest NMO-IgG+ is genetically more similar to systemic lupus erythematosus (SLE) than to MS.

## Results

### Single variant and burden test analysis

In stage I, we performed whole-genome sequencing of 149 NMO cases and 704 controls. After applying a standard next-generation sequencing pipeline and quality control (QC) (Methods, Supplementary Figures 1 and 2), we identified 28,350,499 variants. Most variants were present at low frequencies in 86 NMO cases and 460 controls of European ancestry (Fig. 1, Supplementary Tables  1–5). To increase statistical power, we included additional SNP array genotyping on 144 independent NMO cases and 929 controls (stage II, Supplementary Tables 6–7) corresponding to 129 NMO cases and 784 controls of European ancestry after QC (Fig. 1, Methods). The combined set of 215 NMO cases and 1244 controls gave us 80% of power to detect variants with minor allele frequency (MAF) > 10% and odds ratio (OR) > 2.5. To investigate whether there are genetic differences between AQP4-IgG antibody seropositive and seronegative NMO cases, we stratified our analyses into three categories: AQP4-IgG-seropositive NMO (NMO-IgG+), AQP4-IgG-seronegative NMO (NMO-IgG−), and combined NMO irrespective of AQP4-IgG status (NMO) (Fig. 1).

Given the low prevalence of NMO, we first tested the “Rare Disease–Rare Variant” hypothesis by assessing the accumulation of rare protein-coding variations using gene-burden analysis in 38,134 gene transcripts with at least two rare (MAF < 1%) missense or loss-of-function variants. No genes passed our significance threshold of 2.5 × 10^−6^ (Methods, Supplementary Fig. 3, Supplementary Table 8).

**Fig. 2:**
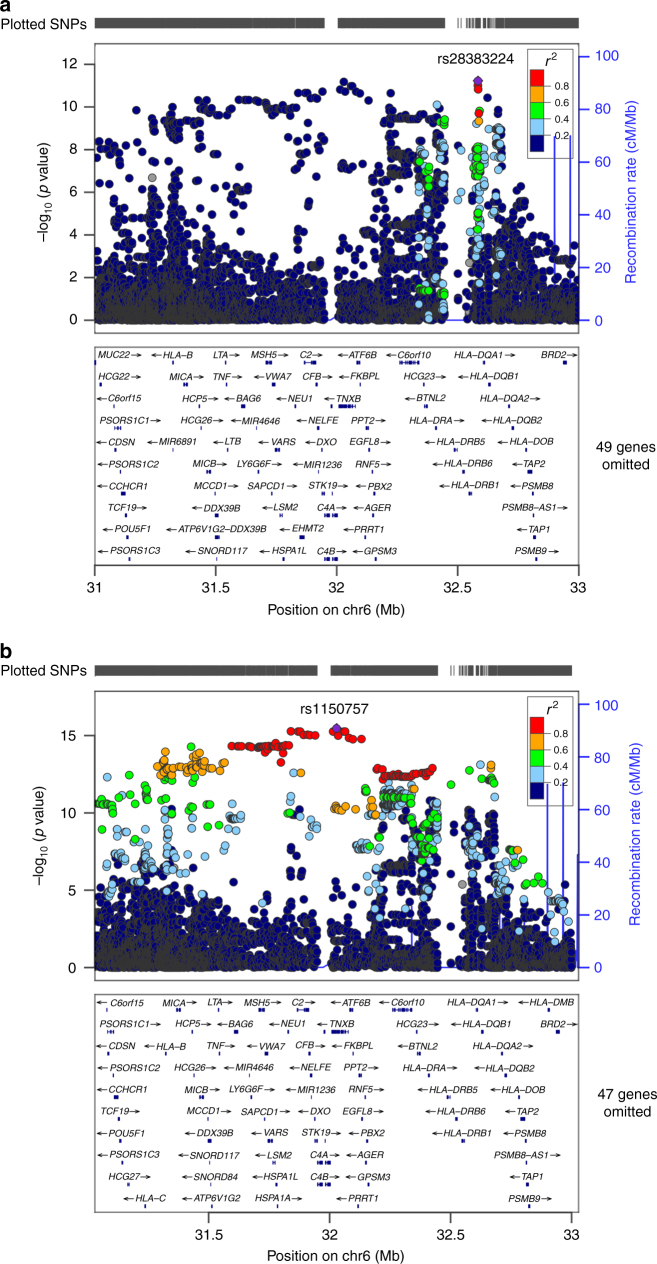
Regional association plots of stage I + II meta-analysis. The *x* axis represents the chromosomal position, and the *y* axis is the –log10 of the *P* value of association. Colors indicate the LD level with the index SNP. Purple lines represent recombination hotspots. **a** Regional association plot of the most significant signal in the combined NMO-IgG+ and NMO-IgG− represented by rs28383224 and closest to HLA-DQA1. **b** Regional association plot of the most significant signal in NMO-IgG+ alone represented by rs1150757 and close to the region mapping to *C4A* and *C4B*

Second, we tested the hypothesis of common variants by conducting a meta-analysis of single variants. After stringent quality control (Methods), 6,804,788 variants (MAF > 1%) in 215 NMO cases and 1244 controls in the combined analysis were used for single variant analyses with logistic regression adjusted for five principal components, and we then combined the evidence using an inverse variance fixed effects meta-analysis of stage I + II results (Fig. 1, Methods). The most significant signal from this analysis of combined NMO (rs28383224, MAF = 0.42, OR = 2.24, *P* = 5.8 × 10^−12^) mapped 21.5 kb downstream of HLA-DQA1 (Table 1, Supplementary Fig. 4, Supplementary Table 9, Fig. 2a). This association was significant in NMO-IgG+ (AF cases 64%, AF controls 42%, OR = 2.66, *P* = 8 × 10^−11^) but had a weaker nominal association in the NMO-IgG− (AF cases 56%, AF controls 42%, OR = 1.76, *P* = 1 × 10^−3^). Given the smaller sample size of the NMO-IgG− group compared with that of the IgG+ group, it seems likely that this signal is shared by both NMO-IgG+ and IgG− and that the result would be genome-wide significant with a larger sample size (Table 1). This is further supported by the similar level of evidence of association observed for NMO-IgG+ and IgG− in the stage 2 samples (Supplementary Table 9).

**Table 1:** Association statistics for genome-wide significant loci in seropositive NMO (NMO-IgG+, *N* = 132 cases, 704 controls), seronegative NMO (NMO-IgG−, *N* = 83 cases, 704 controls), and the combined data sets (*N* = 215 cases, 704 controls)

							**NMO-IgG+**			**NMO-IgG−**			**Combined**		
**Variant**	**Chr**	**Position (hg19)**	**Gene(s)**	**A1**	**A2**	**Freq Ctrl**	**Freq Case**	**OR (95% CI)**	***P***	**Freq Case**	**OR (95% CI)**	***P***	**Freq Case**	**OR (95% CI)**	***P***
rs28383224	6	32583653	*HLA-DQA1*	A	G	0.42	0.64	2.66 (1.98–3.56)	**8.01e**-**11**	0.56	1.76 (1.25–2.46)	1.05e-3	0.61	2.24 (1.78–2.82)	**5.88e-12**
rs1150757	6	32029205	*C4A, C4B*	A	G	0.10	0.31	4.66 (3.22–6.74)	**3.33e-16**	0.13	1.21 (0.72–2.03)	0.47	0.24	2.86 (1.98–4.14)	**6.62e-12**
Conditioned on rs28383224															
rs1150757								3.48 (2.38–5.08)	**1.24e-10**		0.89 (0.53–1.51)	0.67		2.17 (1.59–2.96)	9.03e-7
Conditioned on HLA*DRB1-03:01															
rs1150757								3.91 (1.93–7.94)	1.59e-04		0.90 (0.36–2.25)	0.82		2.30 (1.31–4.04)	3.61e-3

Chr, Chromosome; hg19, human genome build 19; A1, Allele 1; A2, Allele 2; Ctrl, Normal controls; Freq, Allele frequency of A1; NMO-IgG+, aquaporin 4 IgG seropositive; NMO-IgG−, aquaporin 4 IgG seronegative; Combined, Combined NMO-IgG+ and NMO-IgG− data sets; OR, Odds ratio for each copy of allele 1; 95% CI, 95% confidence interval. *P*-values in bold represent genome-wide significant signals

A different index SNP (represented by rs1150757) was observed on the NMO-IgG+ (Fig. 2b). The AF of this variant was 10% in controls, 31% in NMO-IgG+ (OR = 4.66, *P* = 3.33 × 10^−16^, Fig. 2b and Table 1) but was not associated with NMO-IgG−. Conditional analysis revealed that the association of rs1150757 was independent of rs28383224 identified in the combined NMO analysis (Table 1, Supplementary Fig. 5). The SNP rs1150757, a synonymous variant in the *TNXB* gene, is located 26 kb from the *C4A* and *C4B* genes (Fig. 2b).

### Association with classical HLA alleles

To further characterize the associations in the HLA region, we imputed classical HLA alleles (Methods). The most significant association in the combined analysis mapped to a set of common HLA alleles in high LD that contain the HLA-DRB1*03:01 allele (OR = 2.71, *P* = 1.90 × 10^−12^). The signal was driven by NMO-IgG+ (4.09, *P* = 3.66 × 10^−16^), not NMO-IgG− (OR = 1.30, *P* = 0.26) (Table 2). The HLA-DRB1*03:01 signal was highly correlated (*r*^2^ = 0.7) with the secondary signal identified by rs1150757 and poorly correlated (*r*^2^ = 0.2) with the main signal (rs28383224, Supplementary Fig. 6). HLA-DRB1*03:01 is nominally associated with NMO^6^. It is worth noting that the Kim et al. NMO GWAS did not detect an association signal in the MHC. Based on the proceedings of the International Histocompatibility Workshop and literature compiled in a publicly available HLA frequencies database (http://www.allelefrequencies.net/), HLA-DRB1*03:01 is much rarer in Asian populations than Europeans. The frequency of HLA-DRB1*03:01 is not reported for Koreans, however, it seems likely that DRB1*03:01 is much less common in Koreans than Europeans, and that the Kim et al. study was therefore not sufficiently powered to detect this association. The association of HLA-DRB1*03 and NMO was previously evaluated in a small study and yielded a similar effect size (OR = 3.23, 95% CI: 1.07–9.82)^7^. In our study, with a model adjusting by the most significant HLA allele (HLA-DRB1*03:01), the most significant HLA allele was HLA-DRB1:15:01 (OR = 1.79, *P* = 3.9 × 10^−3^; OR = 2.08, *P* = 5.8 × 10^−4^; OR = 1.96, *P* = 2.96 × 10^−5^ for NMO, NMO-IgG+ and NMO-IgG−, respectively) (Table 2). HLA-DRB1:15:01 is a well-known risk factor for MS^8^.

**Table 2:** Association statistics for classical HLA alleles in seropositive NMO (NMO-IgG+, *N* = 132 cases, 704 controls), seronegative NMO (NMO-IgG−, *N* = 83 cases, 704 controls), and the combined data sets (*N* = 215 cases, 704 controls)

	**Allele frequency controls**	**NMO-IgG+**		**NMO-IgG−**		**Combined**	
**HLA Allele**		**OR (95% CI)**	***P***	**OR (95% CI)**	***P***	**OR (95% CI)**	***P***
HLA-DRB1*03:01	0.12	4.09 (2.91–5.74)	**3.66e-16**	1.30 (0.83–2.05)	0.26	2.71 (2.05–3.57)	**1.90e-12**
HLA-B*08:01	0.11	4.23 (2.98–5.99)	**5.89e-16**	1.26 (0.78–2.03)	0.35	2.72 (2.05–3.63)	**6.29e-12**
HLA-DQB1*02:01	0.13	3.79 (2.72–5.28)	**3.81e-15**	1.29 (0.82–2.02)	0.27	2.58 (1.96–3.40)	**1.12e-11**
HLA-C*07:01	0.15	3.26 (2.36–4.49)	**5.18e-13**	1.38 (0.90–2.09)	0.14	2.38 (1.83–3.10)	**1.06e-10**
HLA-DQA1*05:01	0.23	2.90 (2.13–3.95)	**1.15e-11**	1.41 (0.97–2.06)	0.07	2.20 (1.72–2.82)	**4.62e-10**
Conditioned on HLA-DRB1*03:01							
HLA-DRB1*15:01	0.13	1.67 (1.13–2.47)	9.97e-03	1.96 (1.30–2.96)	1.33e-03	1.79 (1.33–2.40)	1.39e-04
HLA-DRB1*04:01	0.09	0.19 (0.07–0.51)	1.13e-03	0.70 (0.37–1.33)	0.28	0.41 (0.23–0.70)	1.31e-03
HLA-DQB1*06:02	0.12	1.79 (1.21–2.66)	3.90e-03	2.08 (1.37–3.16)	5.82e-04	1.90 (1.41–2.58)	2.96e-05
HLA-DQA1*03:01	0.19	0.35 (0.20–0.60)	1.42e-04	0.80 (0.51–1.27)	0.35	0.54 (0.38–0.77)	7.80e-04
HLA-DQA1*01:02	0.19	1.56 (1.10–2.21)	1.19e-02	1.50 (1.02–2.21)	0.04	1.53 (1.17–2.01)	1.96e-03

NMO-IgG+, NMO-IgG seropositive for aquaporin 4 autoantibodies; NMO-IgG−, NMO-IgG seronegative for aquaporin 4 autoantibodies; Combined, Combined NMO-IgG+ and NMO-IgG− data sets; OR, Odds ratio; 95% CI, 95% confidence interval. *P*-values in bold represent genome-wide significant signals

### Copy number association analysis

**Fig. 3:**
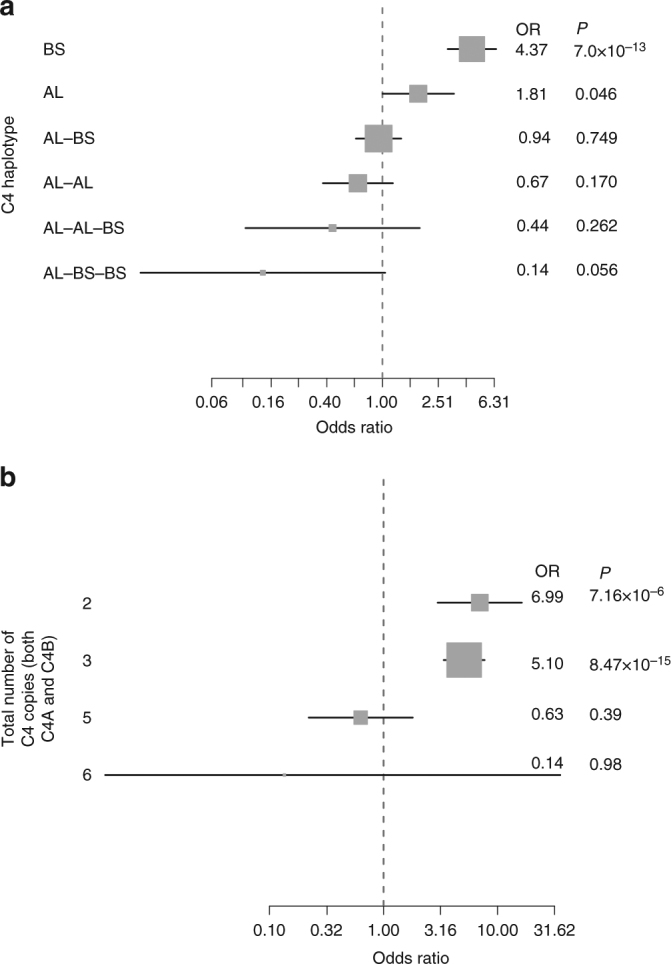
Contribution of C4 copy number to the risk of NMO-IgG+. Odds ratios (ORs) represented by gray squares with sizes proportional to the inverse of their standard errors. Black lines represent the 95% confidence intervals of the ORs. **a** Forest plot demonstrating the association of each the six most common haplotypes in the C4 locus. Abbreviations: BS, haplotype carrying only the short form of *C4B*; AL, haplotype carrying only the long form of *C4A*; AL-BS, haplotype carrying one copy of the long form of *C4A* and the short form of *C4B*; AL-BS, haplotype carrying one copy of the long form of *C4A* and the short form of *C4B*; AL-AL, haplotype carrying two copies of the long form of *C4A*; AL-AL-BS, haplotype carrying two copies of the long form of *C4A* and one of the short form of *C4B*; AL-BS-BS, haplotype carrying one copy of the long form of *C4A* and two copies of the short form of *C4B*. The most common haplotype (one copy of the long forms of *C4A* and *C4B*) was used as a reference (OR = 1) for comparison with the other haplotypes. **b** Forest plot demonstrating the combined effect of total C4 copy number (regardless of size: short or long, and type *C4A* or *C4B*) on risk for NMO-IgG+ . The most common number of total C4 (4) was used as a reference (OR = 1)

To identify potential structural variation associated with NMO, we conducted genome-wide CNV analysis using GenomeSTRiP^9^ on stage I data (Methods). After QC, 22,871 CNV sites were tested for association. A significant association was identified in the NMO-IgG+ group (OR = 6.21, *P* = 1.0 × 10^−9^) for reduced copy number in the region annotated by a complex structural site on the location of complement component 4 (C4), which is located 26 kb from the secondary signal rs1150757 (Figs. 2a, 3a). There was no association of this reduced copy number in NMO-IgG− (OR = 0.17, *P* = 0.17), and therefore the combined analysis showed a reduced effect size and significance (OR = 3.3, *P* = 1.2 × 10^−6^) as compared with the NMO-IgG+ analysis.

Human C4 is encoded by two paralogous genes *C4A* and *C4B*; both exist in a tandem array in the MHC class III region. The most common haplotypes contain one to three *C4* genes (*C4A* and/or *C4B*). The paralogous C4A and C4B genes both have a polymorphic human endogenous retrovirus (HERV) insertion, the presence of which distinguishes long (L) from short (S) forms and allows C4 genes to be subcategorized into four C4 gene “types”: C4AL, C4AS, C4BL, and C4BS^10^. A method has recently been described to characterize the different *C4A/C4B* haplotypes using genetic data (Methods)^10^. We applied these methods in our stage I and stage II data sets and estimated the risk for NMO and its subtypes using haplotype analysis (Methods). The most common haplotype characterized by one copy of the long form of *C4A* and one copy of *C4B* (AL-BL) is present in 40% of the population and was used as a reference. In this analysis, the *C4* BS haplotype—which carries only a single *C4* gene (a short form of C4B)—was the haplotype most associated with NMO-IgG+ risk (OR = 4.37, *P* = 7 × 10^−13^), followed by another single-gene haplotype which also carries only a single *C4* gene (AL) (OR = 1.81, *P* = 0.049) (Fig. 3a). The *C4* BS haplotype was associated with the same magnitude of NMO-IgG+ risk on both Stage I and Stage II studies (Supplementary Fig. 7).

We evaluated the combined effect of C4 copy number (regardless of C4 type and short or long form, i.e., total C4) on NMO-IgG+ risk. As expected, lower than normal total C4 (CN = 2 or CN = 3) was associated with higher risk for NMO-IgG+ (OR = 6.99, *P* = 7.20 × 10^−6^; OR = 5.10, *P* = 8.47 × 10^−15^ for CN = 2 and CN = 3 respectively compared with CN = 4). Compared with the most common total C4 copy number (4), higher total *C4* copy number (*n* > 4) tended (OR = 0.63, *P* = 0.39) to be protective for NMO-IgG+ risk (Fig. 3b) without reaching statistical significance. The protective effect of higher *C4* copy number was only significant for the *C4A*-specific copy number: having three copies of *C4A* compared to two copies was protective for NMO-IgG+ (OR = 0.30, 95% CI (0.14–0.65), *P* = 0.001, Supplementary Fig. 8).

### Conditional and Mendelian randomization analyses

**Fig. 4:**
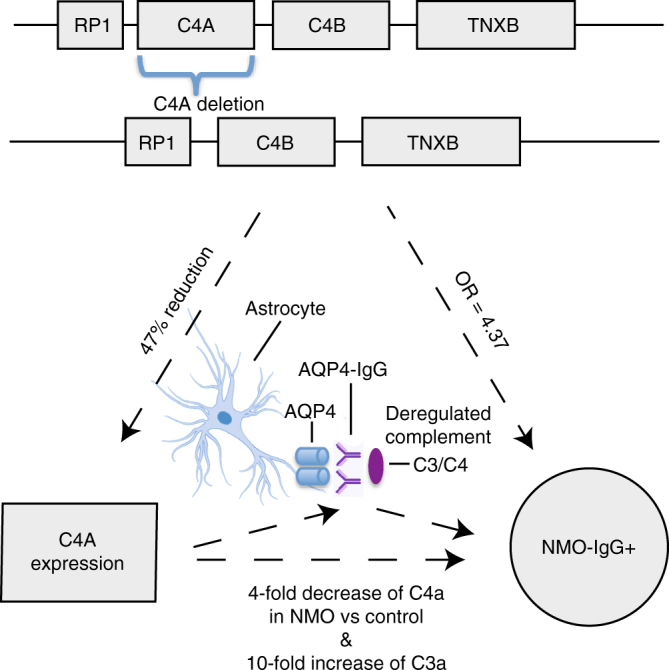
Evidence from human genetics for C4 as a functional role on NMO-IgG+ pathogenesis. Figure representing the main lines of evidence converging on *C4A* deletion as the most likely functional driver for the association with increased risk for NMO-IgG+. Three vertices of the triangle represent observations on (1) DNA variation, (2) gene expression, and (3) phenotype. The sides of the triangle represent the connection between the mentioned vertex. Each box at the top of the figure represents genes near *C4A* and *C4B*. Lines between genes are intergenic regions, in the second row it is depicted the effect of losing one copy of CA4 at the DNA level. More complex rearrangements with lower frequencies ( < 1%) in the general population have been described but are omitted here for simplicity. The 47% reduction in *C4A* expression for each *C4A* deleted gene was estimated in brain tissue^10^. The fourfold decrease of C4a and the 10-fold increase of C3a in NMO vs. the control were measured in serum^11^

We performed additional analyses to uncover the most likely driver of this secondary signal in the MHC locus. The SNP rs1150757, representing this signal, was highly correlated to reduced *C4* gene copy number (*r*^2^ = 0.60). In a multivariate analysis including rs28383224, rs1150757, C4 “deletion” (having fewer than the modal four copies) and HLA-DRB1*03:01, the most significant genetic factor was *C4* deletion (*P* = 5.0 × 10^−4^) followed by rs1150757 (*P* = 0.02); HLA-DRB1*03:01 was no longer significant (*P* = 0.94) (Supplementary Tables 10–12). Using gene expression data from multiple tissues, we found that the SNP correlated with *C4* gene copy number (rs1150757) was significantly associated with C4A expression in many tissues, including nerve and brain (Methods, Supplementary Figs. 9–10). Lower *C4A* copy number also has been shown to correlate with a lower C4A expression in the brain^10^. Furthermore, NMO-IgG+ patients have fourfold reduced C4a protein serum levels compared with the levels in healthy controls^11^. Thus, the cumulative evidence suggests that *C4* deletions may be the functional driver of the association with NMO-IgG+ (Fig. 4). Interestingly, the same *C4* CNV and the HLA-DRB1*03:01 haplotype are associated with SLE^12,13^.

**Fig. 5:**
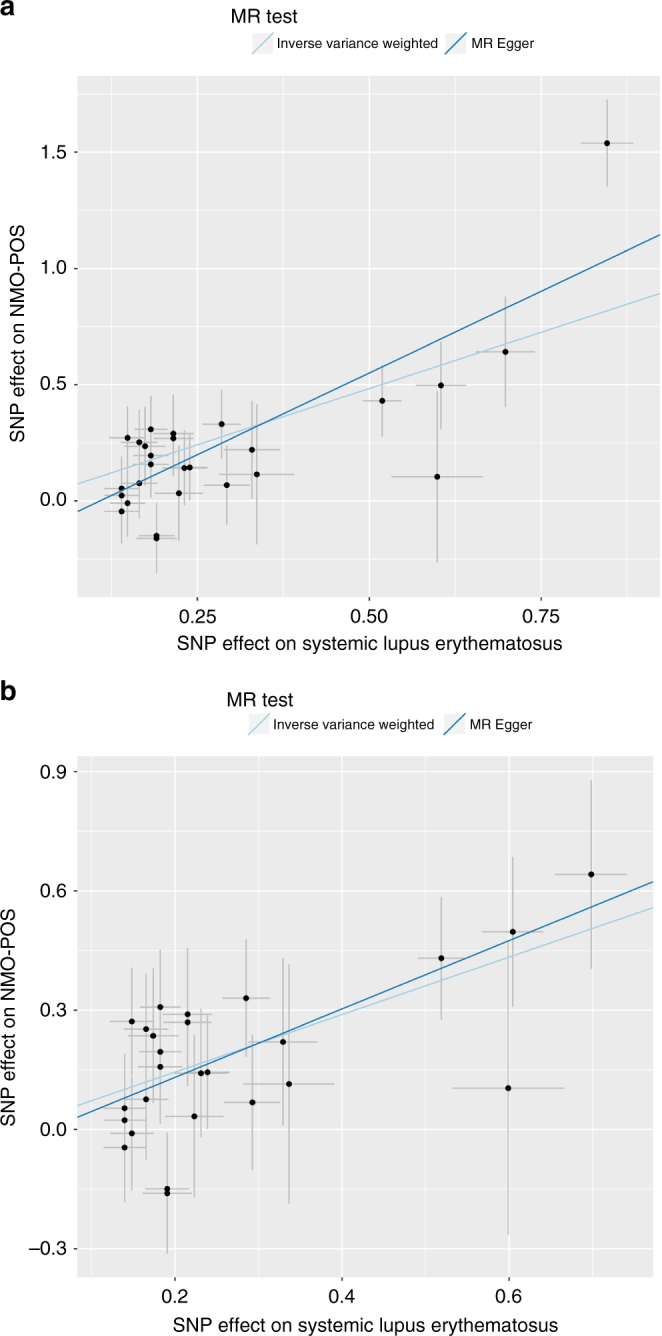
Mendelian randomization analysis of Systemic Lupus Erythematosus (SLE) on NMO-IgG+. Filled dots represent effect size for SLE (*x* axis) and NMO-IgG+ (*y* axis) for a given SLE signal. Error bars reflect s.e.m. Light blue line represents the inverse variance weighted MR effect of SLE on NMO-IgG+. Dark blue line is the MR Egger effect of SLE on NMO-IgG+. **a** MR effects on all genome-wide significant SLE variants on NMO-IgG+. **b** MR effects on all genome-wide significant SLE variants on NMO-IgG+ after excluding rs1150757, the proxy SNP for the C4 copy number variation

**Fig. 6:**
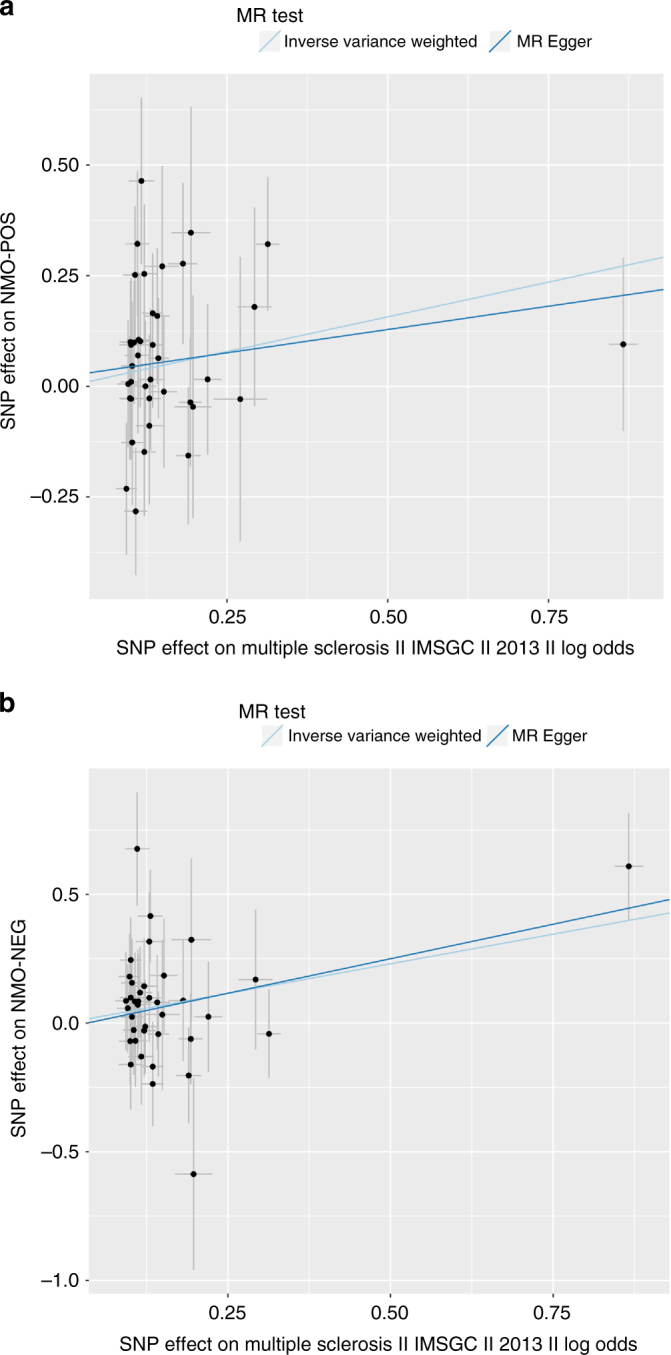
Mendelian randomization analysis of multiple sclerosis (MS) risk variants on NMO. Filled dots represent effect size for MS (*x* axis) and NMO (*y* axis) for a given MS signal. Error bars reflect s.e.m. Light blue line represents the inverse variance weighted MR effect of MS on NMO. Dark blue line is the MR Egger effect of MS on NMO. **a** MR effects on all genome-wide significant MS variants on NMO-IgG+. **b** MR effects on all genome-wide significant MS variants on NMO-IgG−

To evaluate the shared genetic risk factors of SLE and NMO further, we performed a two-sample Mendelian Randomization test (MR, Methods) using 30 genome-wide significant markers for SLE^14^ to evaluate the causal effect of SLE on NMO-IgG+. MR predicted a linear causal effect of SLE markers on NMO-IgG+ (*B*_MR_ = 0.96, *P*_MR_ = 5.66 × 10^−15^; Fig. 5a). There was evidence for pleiotropy (i.e., at least one genetic variant was associated with NMO-IgG+ as well as well as SLE) (*P*_MR_pleiotropy_ = 0.02) driven by rs1150757 as confirmed by a leave one out sensitivity analysis (Supplementary Fig. 11). SLE markers were still predictive for NMO-IgG+ even after excluding the proxy variant of the C4 CNV (rs1150757) (*B*_MR_ = 0.73, *P* = 5.35 × 10^−10^; Fig. 5b). The MR analysis for SLE markers on NMO-IgG− was weaker but still significant (*B*_MR_ = 0.31, *P*_MR_ = 0.02). Next, we evaluated the causal link between MS and NMO. Interestingly, the MR effect of known MS genetic variants in NMO-IgG+ was much weaker than the MR effect of known SLE genetic variants in NMO-IgG+ (*B*_MR_ = 0.31, P_MR_ = 0.02, Fig. 6a). In contrast, the MR effect of MS loci was stronger than the SLE effects on NMO-IgG− (*B*_MR_ = 0.46, *P*_MR_ = 0.004, Fig. 6b). These data suggest that biological risk factors for NMO-IgG+ are more closely linked to those of SLE than those of MS, an interesting finding as before the discovery of the AQP4 antibodies, NMO was considered a sub-type of MS.

## Discussion

Persistence of cellular debris along with impaired killing of compromised cells are proposed to stimulate chronic inflammation in interferon-producing myeloid dendritic cells, which drive B-cell autoimmunity in SLE patients with complement component deficiency^15^. SLE-associated autoantibodies target damaged cell components, such as dsDNA and ribonucleoproteins. AQP4, the target of the NMO autoantibodies, was increased twofold in an animal model of muscle after exercise^16^. Thus, one could hypothesize that AQP4 autoantibodies arise in NMO patients because of the tendency towards reactivity to damaged cell components, similar to autoantibody production in SLE. Consistent with this hypothesis, AQP4-IgG-seropositive NMO patients are threefold more likely to develop additional non-organ-specific antibodies than AQP4-IgG-seronegative NMO patients^17^.

Alleles of the *C4A*/*C4B* locus that increase expression of *C4A* have previously been found to increase risk for schizophrenia^10^. In NMO, we found evidence for association with *C4A* copy number, and a non-significant trend toward association of *C4B* (Fig. 3a, Supplementary Fig. 7); for both genes, increased copy number tended to be protective. A potential model is that both *C4A* and *C4B* offer some protection from NMO, but that *C4A* is more strongly protective than *C4B* is. Biochemically, *C4A* and *C4B* differ at the site that determines what molecules they opsonize in tissues^18,19^; genetically, the relative strengths of association of *C4A* and *C4B* (with NMO and schizophrenia) might reflect the encoded proteins’ differential binding to relevant sites in each tissue.

In summary, we have made several relevant observations: (1) The most significant genetic marker for NMO (rs28383224) was shared between NMO-IgG+ and NMO-IgG−, suggesting that at least one shared genetic determinant exists for both conditions. Data from the GTEx project (Methods) do not suggest that rs28383224 regulates C4 expression, and the mechanism of action of rs28383224 in NMO risk remains unknown. (2) *C4* CNV was associated with NMO-IgG+ but not NMO-IgG−, providing a potentially novel biological hypotheses for seropositive NMO pathogenesis related to low C4 expression. Given the complexity of the MHC region, larger studies will be needed to finemap and disentangle the role of classical HLA alleles to that of *C4* structural variation as well as to understand the effect of these haplotypes on the NMO-IgG negative subgroup. (3) The association of the same C4 deletions in SLE and NMO-IgG+ and the genetic link observed in the Mendelian Randomization study between SLE and NMO-IgG+ suggest a potential common strategy for therapeutic targeting of autoantibody production. Interestingly, both diseases also demonstrate separate roles for complement-mediated effector mechanisms involving the later elements in the cascade^20^. The associations of *C4* duplication with schizophrenia highlight the remarkably pleiotropic biology of the complement system and indicate that its genetic variation will yield a rich trove of insights into disease mechanisms.

## Methods

### Stage I samples

De-identified samples were obtained from the University of Texas Southwestern and the Accelerated Cure Project. Both organizations stored blood specimens under IRB-approved protocols as part of longitudinal studies of NMO. Informed consent for broad genetic research was obtained, this project was approved by the UT Southwestern IRB board. Patients consented for their specimens to be stored and used in future genetics studies. We used 2006 NMO diagnostic criteria^21^ that require optic neuritis and transverse myelitis plus two of the following three supportive elements: (1) longitudinally extensive lesions ( ≥ 3 vertebral segments in length); (2) magnetic resonance imaging of the brain with normal findings or with findings not consistent with MS; and (3) NMO-IgG seropositivity.

Anti-AQP4 serostatus was determined via standardized assays, including enzyme-linked immunosorbent assay (ELISA) or cell-based assay. ELISA-based detection was obtained from one of the numerous labs that offer the test (i.e., Mayo Clinic, Quest, Labcorp, Athena). CBAs were obtained from the Mayo Clinic Labs. This assay utilizes cells transfected with AQP4 and detects antibody binding using flow cytometry to detect the presence of anti-AQP4 antibodies. DNA was extracted from 149 NMO cases (124 NMO-IgG+ /25 NMO-IgG−). In total, 21/149 cases were males. The mean age was 45.5 years (Supplementary Table 1).

The control sample consisted of a cohort of 704 samples of European ancestry from the Genomic Psychiatry Cohort^22^ sequenced as part of the InPSYght project. The Broad Institute certified that an Institutional Review Board reviewed and verified that DNA used for scientific analyses and data generated or shared for limited research purposes are consistent with the informed consent of study participants from whom the samples were obtained. This cohort contains matched schizophrenia and bipolar disorder cases and controls. In total, 460/704 samples in the control cohort were males.

### Stage II samples

In the second stage, we obtained DNA from 144 NMO cases (78 NMO-IgG+ / 68 NMO-IgG−) from an extension of the Accelerated Cure Project using the same protocols and diagnostic criteria as the original ACP discovery samples (see above). Informed consent for broad genetic research was obtained, this project was approved by the UT Southwestern IRB board. The mean participant age was 46 years.

In total, 929 neurologically healthy controls of European ancestry were obtained as controls from the following Coriell projects: NDPT020, NDPT079, NDPT082, NDPT084, NDPT090, NDPT093, NDPT095, NDPT098, and NDPT099. These projects contain samples of unrelated individuals of European ancestry collected from North America without neurological disorders. Of these individuals, ~ 50% are female. The ages at collection ranged from 56 to 95 years. The data collected on these subjects included Control Clinical Data Elements as outlined in the Coriell NINDS Genetics Repository guidelines and detailed medical and family histories. All participants were specifically asked about the following disorders, which were not present: Alzheimer’s, amyotrophic lateral sclerosis, ataxia, autism, bipolar disorder, cerebrovascular disease, dementia, dystonia, Parkinson’s, and schizophrenia. None of the patients had a first-degree relative with one of the known primary neurological disorders mentioned above. All samples and data were collected with informed consent under local IRB-approved protocols.

### Stage I whole-genome sequence data processing

Sequence reads were processed and aligned to the reference genome (hg19) with the BWA algorithm and processed with Picard (http://picard.sourceforge.net). Polymorphic SNP and indel sites and genotypes were called with the HaplotypeCaller from GATK v3.1^23–25^. The HaplotypeCaller algorithm is an assembly-based method that determines genotype likelihoods independently in each sample and then jointly considers data from all samples in the cohort to make confident genotype calls and filter low-quality sites^26^.

### Stage II SNP array genotyping and data processing

Stage II samples were genotyped using the Affymetrix Axiom Biobank microarray with a standard protocol. Genotypes were called in the combined cases and controls data set using Affymetrix Power Tools, version 1.18.0.

### Callsets and QC whole-genome sequence data in stage I

The raw callset contained 50,285,563 variants (20,644,156 singletons) in 853 samples. Biallelic SNPs represented 86.2% (43,322,390) of the raw callset, whereas small INDELs (4,783,485) and multiallelic variants (2,179,688) represented 9.5 and 4.3%, respectively. Variant quality was evaluated and filtered using the GATK’s Variant Quality Score Recalibration (VQSR) approach^25^. After VQSR, the callset contained 46,672,452 passing variants (99.2% non-coding), of which 19,595,281 were singletons.

In addition, we applied the following sample QC filters: 200 genetic outliers (54 cases, 146 controls), which were three or more standard deviations away from the population mean of the first eight principal components (Supplementary Fig. 1). In the genetic outliers of the case series, we detected 28 African-Americans, 10 south Asians, and 10 Asians. The remaining samples represented individuals of northwestern European ancestry.

Ten additional samples (six cases, four controls) had IBD PI_HAT > 0.4 consistent with first-degree relationships. Other sample QC filters included samples with abnormally high numbers of non-reference alleles or heterozygosity or with abnormally low call rates or low concordance with prior SNP array genotypes. A breakdown of these and other sample QC filters is provided in Supplementary Table 2. After this step, we recomputed variant metrics and excluded monomorphic variants (Supplementary Table 3).

The clean callset contained 545 samples (86 cases and 460 controls) and 29,092,408 variants, of which 12,851,416 were singletons. Aggregated and per sample statistics are displayed in Supplementary Tables 4 and 5, respectively. There were no significant differences in cases and controls in singletons and het calls after QC+, but there was still a significant difference in call rate derived from a difference in coverage (Supplementary Fig. 2).

### Callsets and QC SNP array in stage II

The raw callset contained 144 cases and 929 controls. Genotypes were refined using the Affymetrix Axiom Best Practices Genotyping Analysis Workflow. Only variants passing the variant QC Affymetrix best practices implemented in the software SNPPolisher were included^27^. A detailed list of sample and variant QC filters is available on the Supplementary Tables 6–7.

Additional sample and variant QC was performed using PLINK 1.9^28^. In short, twelve samples with mismatches between the genetic and reported genders were removed. Five samples failed the sample genotyping rate (missing rate > 5%) and were removed. An additional three were removed based on principal component analysis to retain only samples of European ancestry. Finally, eight samples with identities by descent > 0.40 with other samples were removed. In total, 11,758 markers with duplicated rsID and/or with duplicated chromosomal positions were excluded. An additional 25,988 variants did not pass the genotyping call rate and were excluded. A total of 24,678 variants with a differential missing genotype rate *P* < 0.05 between cases and controls were excluded from further analyses. the final cleaned data set contained 638,524 variants in 129 cases and 784 controls.

Before imputation with the Haplotype Reference Consortium (HRC)^29^, we excluded variants using the following thresholds: 691 based on a HWE *P* < 5 × 10^−6^, 938 variants with genotyping rate < 5%, 1073 variants with MAF < 0.1% and 37,603 variants of A/T or C/G genotypes. Variants were then matched to the strand reported by the HRC release 1–1. A total of 39,131,578 variants were imputed on the autosomal chromosomes. The imputation *r*^2^ of the top signals (rs28383224 and rs1150757) was 1.0.

### Burden test association analysis

For burden analysis, we grouped variants based on gene annotations. Two main categories were used: non-synonymous and loss-of-function variants. For each of these categories, we applied two MAF filters (1 and 5%). Only variants with call rates > 50% were included. Genes with at least two variants in the mentioned categories were analyzed. Five principal components were used as covariates using the Logistic Wald Test implemented as b.collapse in the EPACTS package. We used a significance threshold of 2.5 × 10^−6^ to account for ~ 25,000 protein-coding genes in the human genome.

### Single variant association analysis

Variants with MAFs > 1% were selected for single-variant association testing using a Wald test implemented in the software package EPACTS. To further minimize batch effects, we applied the following additional filters to minimize the amount of false positive associations: *P* missing < 0.05, *P* HWE < 5 × 10^−8^. After these filters, 7,828,420 and 7,495,108 variants were individually tested for associations on the stage I and II data sets, respectively. We used a logistic model to estimate significance, including five principal components as covariates. In addition to analyzing combined cases, we performed stratified analyses based on the anti-AQP4-IgG status.

### Meta-analysis of single variant associations

An inverse variance fixed effects meta-analysis was run using METAL^30^ on all 6,804,788 shared variants in stages I and II. For the imputed data, an additional filter using an imputation quality score of RSQ > 0.30 was applied. The standard *P* value threshold for common variants of *P* < 5 × 10^−8^ was used as the threshold for significance. Inflation factors were marginal: 1.036, 1.027, 1.036 for Combined, NMO-IgG−, and NMO-IgG+, respectively. These genomic inflation factors were used to adjust test statistics.

### Conditional analysis

After identifying the first signal, a conditional analysis on the top hit (represented by the variant rs1150753) was conducted using the genotypes from that variant as an additional covariate in a logistic regression implemented in EPACTS.

### HLA imputation and association

We imputed classical HLA alleles using the method implemented in the SNP2HLA v 1.0 package^31^, which uses Beagle v 3.0.4 as the imputation machine^28^ and the T1DGC reference panel. Up to 424 HLA alleles were imputed of which 190 had a MAF > 1% in the following HLA genes: The HLA genes: HLA-A, HLA-B, HLA-C, HLA-DPA1, HLA-DPB1, HLA-DQA1, HLA-DQB1, HLA-DRB1.

We found that 90.3% of the BS haplotypes traveled together with the HLA-DRB1*03:01 allele. Conversely, 78.3% of all the HLA-DRB1*03:01 haplotype carried the BS haplotype. We found in total 126 haplotypes that had informative recombinations (having either HLA-DRB1*03:01 or BS but not both).

HLA alleles were tested for association using the genotype probabilities from beagle as input and the five first principal components as covariates in a logistic regression implemented in Plink.

### CNV calling on whole-genome sequencing data

We discovered and genotyped CNVs in the Stage I WGS data using the CNV pipeline of Genome STRiP^32^. In brief, Genome STRiP’s CNV pipeline genotypes segments of the genome using observed read depth-of-coverage by fitting a Gaussian Mixture Model constrained to call integer copy numbers for each segment. Read depth counts are normalized for the overall sequencing depth and observed GC bias of each library. Segments are iteratively adjusted to find the strongest model fits, and segments in which one or more samples are strongly genotyped as having a copy number differing from the expected ploidy are emitted. We filtered out deletion and mixed deletion/duplication sites under 1 kb in length and duplications under 2.5 kb.

### C4 paralog and haplotype analysis

Association analysis of the CNV calls produced by Genome STRiP revealed a strong association with a CNV adjacent to the C4 gene in the MHC region of chromosome 6. Recent work showed that the C4 locus occurs as complex structural haplotypes and that specific structural haplotypes are associated with risk for schizophrenia^10^. In brief, each haplotype can contain a variable number of copies of each of two paralogs of the C4 gene, designated *C4A* and *C4B*. Furthermore, each paralog can be found in either a long form or a short form, depending on the presence or absence of a HERV insertion. The most common haplotypes among European ancestry populations are AL-BL (indicating a haplotype with both a long form of the “A” paralog and a long form of the “B” paralog), AL-BS, AL-AL, and BS.

To determine total copy numbers of each paralog of C4, we used the segmental duplication genotyping procedure in Genome STRiP (CNV Discovery Set 2)^32^, using a set of custom intervals diagnostic for the C4 and C4-HERV copy numbers. Reads aligning to the critical regions of exon 26 that distinguish *C4A* from *C4B* were counted and likelihoods for the *C4A* to *C4B* ratio were determined under a binomial model conditional on the total C4 copy number.

We then determined the likelihood of C4 haplotypes with the following procedure. Given the likelihood of diploid copy numbers for each segment (*C4A*, *C4B*, HERV) generated by previous step, we applied the procedure described in Sekar et al. (2016)^10^ to determine the likelihood of each haploid copy number for each segment, taking into account the estimated frequency of each haploid copy number in the sampled population using an expectation-maximization algorithm. We then assigned likelihoods to each of the possible structural C4 haplotypes defined by Sekar et al. (2016)^10^ based on the haploid copy number genotype likelihoods across the three segments. We started by examining the diploid copy numbers across the three sites and finding all combinations of haplotypes that could explain them. For example, if the diploid copy numbers are *C4A*:3, *C4B*:1, HERV:3, then the structural haplotypes AL-AL and AL-BS are a potential combination. Each structural haplotype was given likelihood equal to the product of site haploid copy number likelihoods. We also assigned a small amount of likelihood to an < UNK > haplotype allele to account for unknown haplotypes or errors in genotyping.

Finally, we used Beagle’s genotype refinement capability to assign the most likely structural haplotype genotypes to each sample. For this step, we used the SNPs defined in the imputation panel in Sekar et al., lifted over to hg19 coordinates, refining all data from samples from Stage 1 cases and controls simultaneously.

### Imputation of C4 structural alleles in stage II data

We imputed C4 structural alleles into the Stage II genotype data using Beagle. We used an imputation reference panel (Kamitaki, Handsaker, et al., in preparation) consisting of genotypes taken from whole-genome sequencing data for a multi-ethnic cohort of 1265 individuals from the Genomic Psychiatry Cohort^22^ sequenced as part of the InPSYght project. C4 structural haplotypes were determined using a methodology similar to that described above. For imputation, we used the same set of SNPs described above.

### Haplotype association analysis

Phased C4 structural alleles from the CNV calling were used in a haplotype analysis to understand the effect of the different C4 alleles in relation to NMO-IgG+. The most common haplotype (AL-BL) was used as reference for comparison to the other haplotypes.

### Total C4 dosage analysis

For each participant, we used the CNV data to count the total number of C4 alleles (*C4A* + *C4B*) and use that as predictor for NMO-IgG+. The most common combination (C4*n* = 4) was used as a reference for comparison in a logistic regression using the other combinations as factors with five principal components as covariates. Statistical analyses were conducted using the R statistical software package.

### CNV imputation quality assessment

To assess the accuracy of our computational methods for determining C4 copy numbers from whole-genome sequencing data, phasing the copy numbers into C4 structural haplotypes, and imputing C4 structural haplotypes based on genotyping data, we used a set of 111 HapMap CEU samples. The C4 structural haplotypes of these samples were fully characterized by Sekar et al.^10^. using a combination of trio phasing and verification of integer copy numbers with digital droplet PCR (ddPCR). Although we do not have high-coverage sequencing data for these samples, we were able to use low-coverage (~ 4×) sequencing data for 87 of these samples from the 1000 Genomes project and SNP genotypes from the HapMap project to validate several of our methods.

A key step in our methods for inferring phased C4 structural alleles is determining the total diploid copy number of C4, the C4 HERV element that distinguishes long from short forms of the C4 gene, and *C4A* and *C4B* paralog-specific variants. We applied our methods for assessing total C4 copy number to the HapMap low-coverage sequencing data described above and evaluated the results against the validated C4 structural haplotypes for these samples. The results are shown in the Supplementary Table 13. Our methods assigned the correct total C4 copy number to 86/87 samples, with a 98.9% concordance rate with the validated C4 haplotype data. We were also able to accurately call the total number of C4 HERV elements, indicating the total copy number of long forms of the C4 gene, in 88.5% of the samples. Our method struggled to call paralog-specific copy number of *C4A* and *C4B* in this low-coverage data, likely due to the low expected number of reads at the sites of paralog-specific variation necessary to distinguish between the two forms. However, based on the performance of our phasing and imputation pipeline described below, we believe that the paralog-specific copy number performance is much better in high-coverage sequencing data.

We also used the HapMap samples to evaluate the imputation pipeline used in the replication phase of this study. We took the paralog-specific copy number estimates from the high-coverage WGS data for NMO cases and controls in the first phase of the study and combined them with genotype data from those samples as well as the published SNP genotypes for the HapMap samples. We then imputed C4 structural haplotypes into the HapMap samples using the same pipeline that was used in the replication phase of this study. We used only SNP sites present in the HapMap data set. No copy number data from the HapMap samples was used in the imputation pipeline. Evaluating the results against the validated C4 haplotypes from Sekar et al.^10^, we found that we accurately imputed both haplotypes into 90 of the 111 HapMap samples (81.1%), and had a correct imputation of at least one haplotype in 106/111 (95.5%). We found that we were able to impute the dosage of the four most common haplotypes with varying levels of accuracy (Supplementary Table 14). Our relative success at imputing haplotypes into the HapMap samples gives us some confidence that the copy number likelihoods computed from the high-coverage WGS data, including those for paralog-specific copy number estimates, are accurate enough to phase the correct haplotypes in samples involved in the WGS phase of this study.

### Multivariate analysis

We analyzed the independent effects of the three highly correlated genetic factors associated with NMO-IgG+ in the HLA region: the C4 deletion and the presence of the classical HLA allele DRB1*03:01 and the SNP rs1150757 using a multivariate logistic regression implemented in the R statistical package (function glm). C4 deletion was defined as having a combined *C4A* + *C4B* copy number of 2 or 3. A multivariate analysis with the total number of C4 instead of absent/present C4 yielded similar results (*P* = 0.004 for total C4, *P* = 0.009 for rs1150757, and *P* = 0.98 for HLA-DRB1*03:01).

This analysis was conducted both separately on each stage and in combination. In the combined analysis, an additional dummy variable identifying the stage was added. The top five principal components were also included in the multivariate analysis.

### Sensitivity analyses

A sensitivity analysis was performed by excluding all individuals with the HLA-DRB1*03:01 allele in a logistic regression testing effect of the presence/absence of the C4 deletion in 46 NMO-IgG+ and 816 controls. The C4 deletion was still significant in those individuals without the HLA-DRB1*03:01 allele (*P* = 0.007). Conversely, when we restrict the analysis by excluding samples with one or more deletions of C4 in 44 NMO-IgG+ and 920 controls, the HLA-DRB1*03:01 allele is no longer significant (*P* = 0.20).

A second sensitivity analysis was performed by excluding schizophrenia/bipolar disease cases that were included as controls in the discovery set. The results were very similar to the original results (Supplementary Table 15).

### C4 expression data

The rank-normalized expression levels of *C4A* in 43 different tissues were evaluated in up to 361 individuals of the GTEX project (dbGaP Accession phs000424.v4.p1). The genetic variant rs1150757, which is highly correlated with the C4 CNV, was associated with *C4A* expression in all tissues to different degrees. Regression analyses and plots were obtained from the GTEX portal (http://www.gtexportal.org).

### Mendelian randomization analysis

MR analysis was performed using the Two-Sample MR package on 30 SLE and 39 MS genetic markers from previous meta-analysis of genome-wide association studies^8,14^. Markers were pruned to remove LD (*r*^2^ > 0.05) and only markers that could be harmonized in terms of alleles with the NMO GWAS were kept. The following SNPs could not be harmonized for SLE (rs2736332, rs35472514) and MS (rs1131265, rs12296430, rs1359062, rs212405, rs9736016, rs9989735). Inverse variance weighted MR and MR Egger were used to estimate the causal effect of SLE and MS on NMO.

### Data availability

Reasonable requests for the data sets generated and analyzed during the current study are available from the authors of this paper. Summary statistics are available from the Biogen statistical genetics site (https://github.com/Biogen-Inc/statgen) and will be also posted at the NHGRI-EBI GWAS Catalog (https://www.ebi.ac.uk/gwas/downloads/summary-statistics).

## Electronic supplementary material

Supplementary Information
