# Supplementary material for: A whole-genome sequence study identifies genetic risk factors for neuromyelitis optica
Source: Nat Commun. 2018 May 16;9:1929. doi: 10.1038/s41467-018-04332-3 (PMC5955905; doi:10.1038/s41467-018-04332-3)

Supplementary Material

A whole-genome sequence study identifies genetic risk factors for Neuromyelitis Optica

Estrada et al.

Supplementary Tables

Supplementary Table 1. Sample characteristics of NMO cases (Stage I)

	NMO-IgG+ (N=66)			NMO-IgG- (N=20)			NMO-IgG+ vs.
	**Number**	**Mean**	**S.D.**	**Number**	**Mean**	**S.D.**	**NMO-IgG- P-value**
**Age**	65	48.89	14.83	19	43.50	11.61	0.11
**Gender (% Female)**	65	86%		19	74%		0.21
**Type of first symptoms**							
**Visual**	21	33%		7	39%		Reference
**Spinal**	39	61%		7	39%		0.30
**Both**	4	6%		4	22%		0.19
**Smoker (%)**	43	35%		14	64%		0.06
**Years Diagnosed**	65	2.26	2.19	19	2.18	2.39	0.88
**Years Symptoms**	65	5.79	6.12	18	6.47	7.21	0.69
**ANA positive**[1]	4	66%		3	17%		0.01
**Total C4**	64	3.41	0.73	19	3.23	0.64	4.00e-04

[1] ANA measurements only available in 19 samples

NMO-IgG+, aquaporin 4 IgG seropositive; NMO-IgG-, aquaporin 4 IgG seronegative; S.D., standard deviation

Supplementary Table 2. Numbers of NMO cases and controls removed in quality control (QC) filters (Stage I)

Sample QC Filter	Number of cases removed	Number of controls removed	Total number of samples removed
Ancestry outliers (3 S.D. from mean PCA)	54	146	200
First-degree relationship	6	4	10
Call rate < 99%	0	21	21
Concordance with array < 99.5%	0	5	5
Mean allele balance <0.505 or > 0.51	0	20	20
Mean depth < 20X	0	17	17
Transition transversion ratio (T_i/T_v) < 2.13	0	9	9
Minor allele count > 3.4M	30	19	49
het/hom ratio > 6.3	2	84	86
Number of singletons > 40,700	42	17	59
m_alt_qf_ab < 0.598	0	2	2
alt_qf_abm50 < 0.20	0	4	4
Total removed[1]	**63**	**244**	**307**

[1]The total number of subjects removed is lower than the sum of filters as samples overlap between filters.

S.D., standard deviation; PCA, principal components analysis

Supplementary Table 3. Numbers of variants removed in quality control (QC) filters (Stage I)

Variant QC Filter	Number of variants removed
Monomorphic	15,446,631
Log odds of variant quality score (VQSLOD)[1] < 0.4603	2,323,482
Genotype quality (GQ) < 30	1,513,335
0.20 > Heterozygotes allele balance (HET_AB) > 0.80	1,401,479
Mean genotype quality of alternative alleles (MEAN_ALT_GQ) < 40	809,020
Depth < 10	393,752
Missing rate > 0.40	99,950
Standard deviation of the depth (DEV_DP) > 15	37,747
Quality (QUAL) < 30	18

[1]For insertions, deletions and multi-allelic variants

Supplementary Table 4. Variant counts by filtration level in whole genome sequencing study (Stage I)

Variant Class	Raw	Pass	QC+
All	50,285,563	46,672,452	28,350,499
Missense	222,347	208,861	135,917
Nonsense	4,077	3,755	2,483
Synonymous	154,007	146,456	94,551
Noncoding	49,887,362	46,296,213	28,108,153
Singletons	20,644,156	19,595,281	12,633,939

Pass: Variants passing VQSR filer. QC+, Variants passing quality control as detailed in Supp. Table 3.

Supplementary Table 5. Variant counts per sample by filtration level in whole genome sequencing study (Stage I)

Variant Class	Raw	Pass
All	3,458,000 ± 249,200	3,215,000 ± 235,900
Missense	7,888 ± 597.4	6,753 ± 543.3
Nonsense	81.5 ± 8	53.4 ± 6.8
Synonymous	8,503 ± 727	7,803 ± 689.2
Noncoding	3,440,000 ± 247,900	3,200,000 ± 234,600
Singletons	NA	22,380 ± 19,880

Supplementary Table 6. Numbers of samples removed in quality control (QC) filters in the genome-wide association study (GWAS) (Stage II)

Sample QC Filter	Number of Samples Removed
Affymetrix Dish quality control < 0.82	4 controls
Affymetrix sample call rate < 97%	3 cases, 16 controls
Affymetrix Plate QC (mean call rate < 98.5%)	92 controls[1]
Gender mismatch	12 controls
Sample call rate < 95%	5 controls
Principal components (PCs) >3 S.D. away from mean Europeans	2 controls, 1 case
Relatedness (IBD > 0.40)	7 controls, 1 case

[1] One plate

S.D., standard deviation; IBD, identical by descent

Supplementary Table 7. Numbers of variants removed in quality control (QC) filters in the genome-wide association study (GWAS) (Stage II)

Variant QC Filter	Number of Variants Removed
Duplicate markers	11,750
Variant call rate < 95%	25,988
P-value for differential missingness < 0.05	24,678
Variants not passing SNPPolisher (default parameters)	122,519

Supplementary Table 8. List of suggestive signals in the whole genome sequencing burden test analysis of non-synonymous variants with minor allele frequency (MAF) < 1% (Stage I)

Data set	Locus	Number of Variants in Gene	Minor Allele Count	Number of Minor Alleles in Affected	Number of Minor Alleles in Unaffected	Proportion Affected Carriers	Proportion Unaffected Carriers	P	Odds Ratio
NMO	*CHRNB4*	12	27	12	15	0.14	0.03	5.31e-5	6.69
NMO-IgG-	*IL1RL2*	11	17	6	11	0.30	0.02	1.97e-5	15.88
	ACSM5	14	34	6	28	0.30	0.06	2.98e-5	2.42
	ZNF736	6	12	4	8	0.20	0.02	5.96e-5	2.95

NMO, aquaporin 4 IgG seronegative and seropositive NMO datasets; NMO-IgG-, aquaporin 4 IgG seronegative NMO dataset

Supplementary Table 9. Strongest association results by study stage and aquaporin 4 (AQP4) IgG antibody serostatus.[1]

			NMO-IgG+			NMO-IgG-			NMO		
Variant[2] (Locus)	Stage	Allele Freq Controls	Allele Freq Cases	OR (95% CI)	P	Allele Freq Cases	OR (95% CI)	P	Allele Freq Cases	OR (95% CI)	
rs28383224*A (*HLA-DQA1*)											
	I	0.41	0.65	2.82 (1.84-4.32)	1.90e-6	0.48	1.27 (0.66-2.46)	0.47	0.61	2.27 (1.57-3.28)	1.[illegible]
	II	0.43	0.63	2.52 (1.70-3.73)	3.99e-6	0.59	1.97 (1.34-2.90)	6.03e-04	0.61	2.22 (1.66-2.96)	5.[illegible]
	I+II	0.42	0.64	2.66 (1.98-3.56)	**8.01e-11**	0.56	1.76 (1.25-2.46)	1.05e-03	0.61	2.24 (1.78-2.82)	**5.[illegible]**
rs1150757*A (*C4A*,*C4B*)											
	I	0.11	0.33	4.67 (2.78-7.86)	6.36e-9	0.08	0.56 (0.16-1.90)	0.35	0.27	3.12 (1.96-4.95)	1.[illegible]
	II	0.10	0.29	4.64 (2.80-7.70)	2.69e-9	0.14	1.43 (0.81-2.51)	0.21	0.22	2.69 (1.83-3.95)	5.[illegible]
	I+II	0.10	0.31	4.66 (3.22-6.74)	**3.33e-16**	0.13	1.21 (0.72-2.03)	0.47	0.24	2.86 (1.98-4.14)	**6.[illegible]**

[1] I^2, the percentage of variation across studies that is due to heterogeneity rather than chance, was 0 for both variants in Stage I+II for the NMO-IgG+ and Combined datasets; I^2 was 18 and 46 for the HLA-DQA1 and C4A/C4B variants, respectively, in Stage I+II for the NMO-IgG- dataset.

[2] The genetic variants reported above were imputed in Stage II with a MACH RSQ = 1.0. Rs28383224 hg19 position chr6:32,583,653. Rs1150757 hg19 position chr6:32,029,205.

NMO-IgG+, aquaporin 4 IgG seropositive dataset; NMO-IgG-, aquaporin 4 IgG seronegative dataset; NMO, Meta-analysis of NMO-IgG+ and NMO-IgG- datasets; Allele Freq, allele frequency; OR, odds ratio; 95% CI, 95% confidence interval

Supplementary Table 10. Multivariate analysis of highly correlated variants from the peak region of association in the NMO-IgG+ dataset

	Stage I		Stage II		Combined	
**Variant**	**OR (95% CI)**	**P**	**OR (95% CI)**	**P**	**OR (95% CI)**	**P**
C4 deletion	2.89 (1.26-6.65)	0.01	2.85 (1.15-7.07)	0.02	2.88 (1.59-5.24)	**5.09e-4**
HLA-DRB1*03:01	1.64 (0.58-4.67)	0.35	0.63 (0.25-1.61)	0.34	0.98 (0.49-1.95)	0.94
rs1150757*A	1.70 (0.60-4.80)	0.32	3.16 (1.08-9.25)	0.04	2.39 (1.15-4.96)	0.02

OR, odds ratio; 95% CI, 95% confidence interval

Supplementary Table 11. Multivariate analysis of highly correlated variants plus rs28383224 from the peak region of association in the NMO-IgG+ dataset

	Stage I		Stage II		Combined	
**Variant**	**OR (95% CI)**	**P**	**OR (95% CI)**	**P**	**OR (95% CI)**	**P**
rs28383224*A	1.98 (1.20-3.27)	0.01	1.98 (1.08-3.64)	2.47e-3	1.18 (1.43-2.77)	**4.72e-5**
C4 deletion	2.86 (1.22-6.67)	0.02	2.96 (1.90-4.61)	0.02	1.37 (1.55-5.28)	**7.78e-4**
HLA-DRB1*03:01	1.01 (0.33-3.04)	0.99	0.44 (0.15-1.30)	0.09	1.45 (0.31-1.31)	0.22
rs1150757*A	1.87 (0.65-5.37)	0.24	3.01 (1.18-7.71)	0.05	1.46 (1.18-5.19)	0.02

OR, odds ratio; 95% CI, 95% confidence interval. rs28383224 is an independent signal to the C4 deletion

Supplementary Table 12. Squared correlation coefficients of main genetic signals in the HLA region

Variant 1	Variant 2	r^2
HLA-DRB1*15:01	HLA-DRB1*03:01	0.02
HLA-DRB1*15:01	rs1150757*A	0.01
HLA-DRB1*15:01	rs28383224*A	0.21
HLA-DRB1*03:01	rs1150757*A	0.73
HLA-DRB1*03:01	rs28383224*A	0.19
rs1150757*A	rs28383224*A	0.13
rs28383224*A	HLA-DRB1*03:01	0.19

Supplementary Table 13. Proportion of correct genotypes amongst the number of genotypes that were compared, to evaluate the accuracy of C4 total diploid copy number estimates in low coverage (4X) WGS data[1]

	For All Genotype Calls (At Any Confidence Level)[2]	For Genotype Calls at 95% Confidence Level	For Genotype Calls at 95% Confidence Level
**Total C4**	98.9% (86/87)	98.8% (81/82)	100.0% (74/74)
**Total HERV**	88.5% (77/87)	93.9% (46/49)	95.0% (19/20)
**C4A**	65.5% (57/87)	100.0% (1/1)	100.0% (1/1)
**C4B**	66.7% (58/87)	50.0% (1/2)	100.0% (1/1)

[1] Calls were based on low coverage WGS data from the 1000 Genomes project and evaluated against the haplotypes validated by Sekar et al using ddPCR and trio phasing.

[2] Confidence level assigned by Genome STRiP's genotyping algorithm.

Supplementary Table 14. Accuracy of C4 haplotype imputation in HapMap samples[1]

C4 Haplotype	Frequency in HapMap Sample Truth Data[2]	Frequency in HapMap Sample Imputed Genotypes	Imputed[3] Genotype R^2	Posterior Genotype Likelihood[4] R^2
**AL-BL**	92	97	0.726	0.722
**AL-BS**	68	67	0.907	0.906
**AL-AL**	24	27	0.707	0.715
**BS**	16	16	0.859	0.897

[1] The imputation pipeline was given SNP genotypes for the HapMap samples as published by the HapMap project, SNP genotype data for the same sites from the NMO cases and controls in Stage I of this study as computed by the GATK HaplotypeCaller pipeline, and copy number likelihoods for the NMO cases and controls as computed by Genome STRiP from depth of coverage in the WGS data.

[2] C4 structural haplotypes of the HapMap samples, verified with trio phasing and ddPCR by Sekar et al., were used as truth.

[3] Correlation of the dosage of each haplotype across samples in the truth data and in the most likely imputed haplotypes.

[4] Correlation of the dosage of each haplotype across samples with the posterior likelihood of that haplotype being present in each sample as computed by the imputation pipeline.

Supplementary Table 15. Sensitivity analysis of association results before and after removal of individuals with schizophrenia or bipolar that were included in the Stage I study.

	With Schizophrenia and Bipolar						Without Schizophrenia and Bipolar					
	NMO-IgG+		NMO-IgG-		Combined		NMO-IgG+		NMO-IgG-		Combined	
Variant	OR (95% CI)	P	OR (95% CI)	P	OR (95% CI)	P	OR (95% CI)	P	OR (95% CI)	P	OR (95% CI)	P
rs113175152	2.66 (1.98-3.56)	8.01e-11	1.76 (1.25-2.46)	1.05e-3	2.24 (1.78-2.82)	5.88e-12	3.02 (2.2-4.15)	7.56e-12	1.93 (1.38-2.71)	1.34e-4	2.48 (1.95-3.16)	2.23e-13
rs1150757*A	4.66 (3.22-6.74)	3.33e-16	1.21 (0.72-2.03)	0.47	2.86 (1.98-4.14)	6.62e-12	4.94 (3.35-7.29)	8.02e-16	1.31 (0.79-2.16)	0.30	3.04 (2.22-4.16)	4.09e-12
C4 deletion	5.59 (3.78-8.27)	7.70e-18	1.33 (0.82-2.17)	0.24	3.20 (2.36-4.32)	4.26e-14	5.33 (3.52-8.07)	2.61e-15	1.38 (0.84-2.26)	0.20	3.13 (2.27-4.32)	3.51e-12
**Conditioned on rs113175152**												
rs1150757*A	3.48 (2.38-5.08)	1.23e-10	0.89 (0.53-1.51)	0.67	2.17 (1.59-2.96)	9.03e-07	3.42 (2.26-5.18)	5.85e-9	0.92 (0.54-1.56)	0.75	2.13 (1.52-2.99)	1.07e-05
C4 deletion	4.1 (2.72-6.19)	1.90e-11	1.00 (0.6-1.67)	1.00	4.1 (2.72-6.19)	1.26e-07	3.73 (2.4-5.78)	4.34e-9	1.01 (0.6-1.7)	0.97	2.23 (1.58-3.15)	4.55e-06

Supplementary Figures

Supplementary Figure 1. Principal components analysis (OCA) of NMO and control samples. The first two principal components of Stage I and Stage II samples after removal of genetic outliers are plotted anchored to the 1000 Genomes project samples. Samples are color coded based on their origin (see legend).

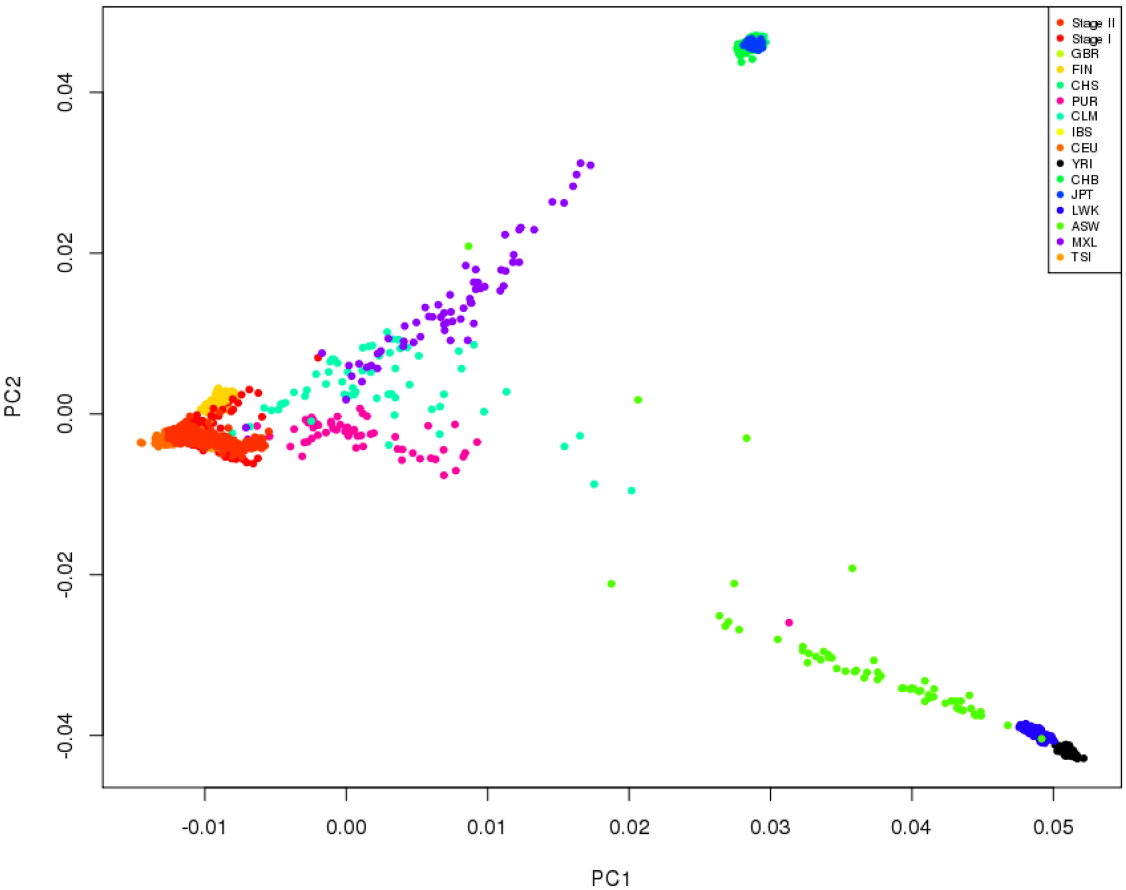

GBR, British in England and Scotland; FIN, Finnish in Finland; CHS, Southern Han Chinese; PUR, Puerto Ricans from Puerto Rico; CLM, Colombians from Medellin, Colombia; IBS, Iberian population in Spain; CEU, Utah residents with Northern and Western European ancestry; YRI, Yoruba in Ibadan, Nigeria; CHB, Han Chinese in Beijing, China; JPT, Japanese in Tokyo, Japan; LWK, Luhya in Webuye, Kenya; ASW, Americans of African ancestry in southwestern USA; MXL, Mexican American in Los Angeles, USA; and TSI, Toscani in Italy.

Supplementary Figure 2. Stage I sample properties after Quality Control (QC).

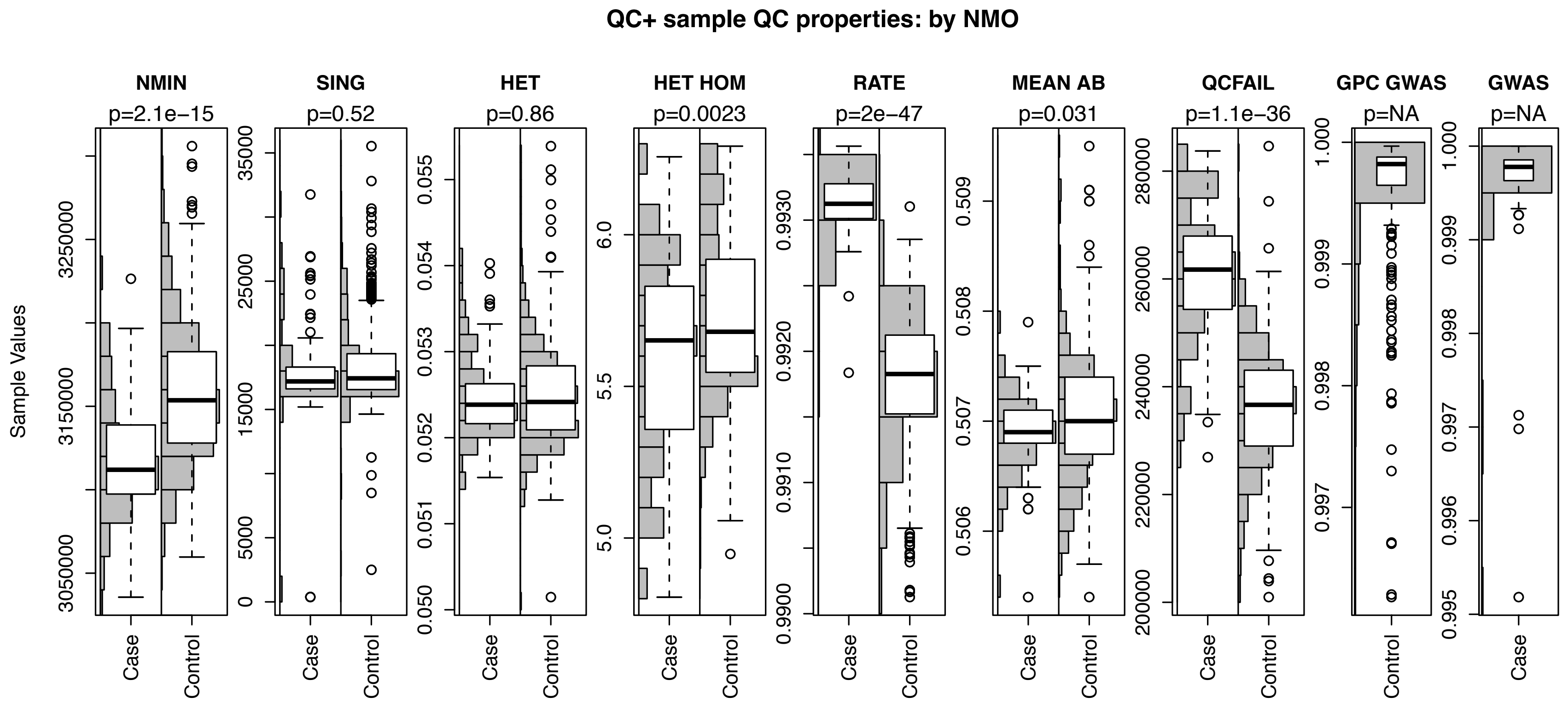

NMIN, Number of genotypes with a minor allele*;* SING, Number of singletons; HET, Proportion of heterozygote counts; HET HOM, Heterozygotes/Homozygotes ratio; RATE, Genotyping rate for individual; MEAN AB, Mean Allele Balance; QCFAIL, Number of QC failing variants; GPC GWAS, Non-reference genotype concordance in controls; GWAS, Non-reference genotype concordance in cases.

Supplementary Figure 3. Quantile-quantile (QQ) plots for gene burden tests (Logistic Wald test) on non-synonymous variants with a minor allele frequency <1% in the whole genome sequencing study (Stage I). **A.** NMO-IgG+. **B.** NMO-IgG-. **C.** Combined NMO-IgG+ and NMO-IgG- data sets.

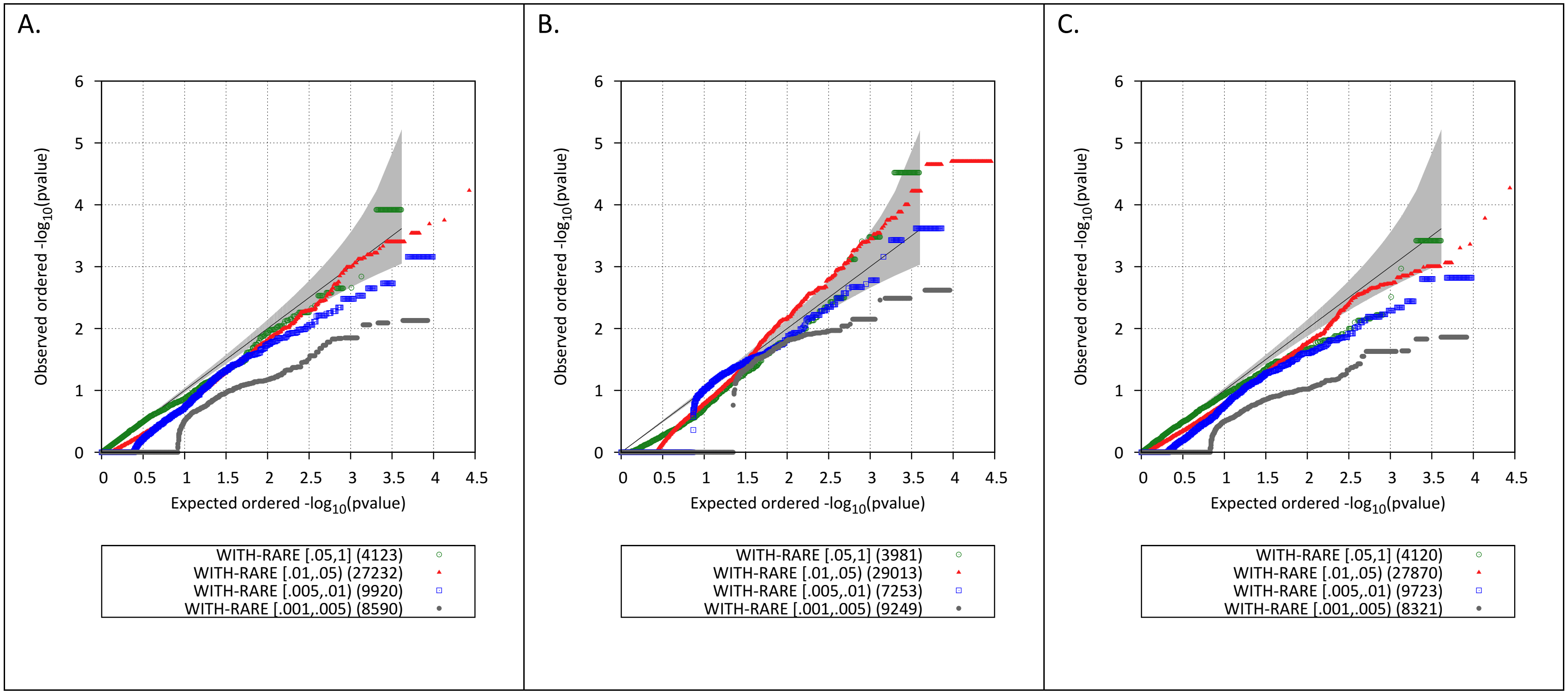

Supplementary Figure 4. Quantile-quantile (QQ) and Manhattan plots for the NMO genome-wide association study (GWAS) meta-analysis (Stage I+II). A. NMO-IgG+. **B**. NMO-IgG-. **C.** Combined NMO-IgG+ and NMO-IgG- data sets.

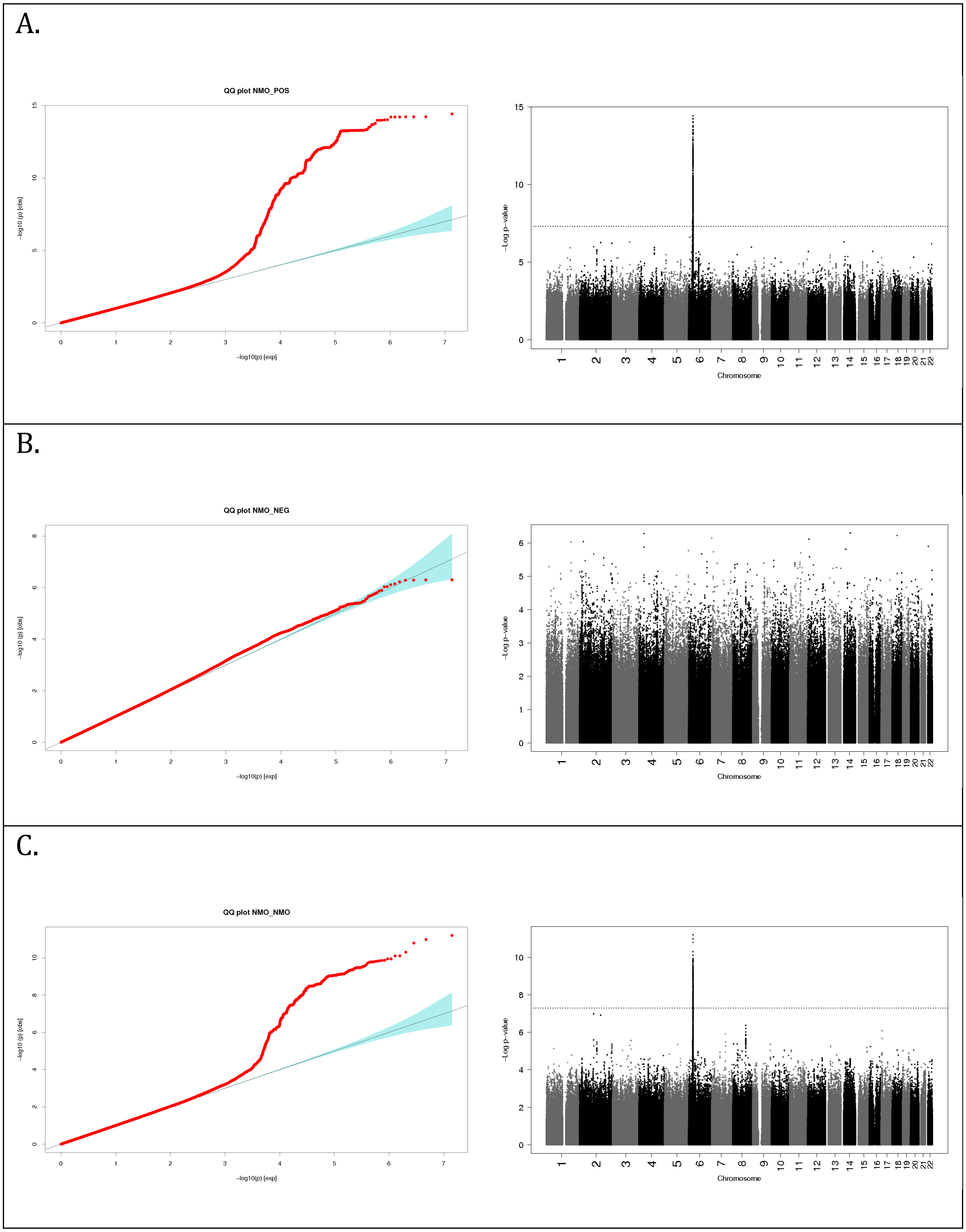

Supplementary Figure 5. Regional association plot of the most significant variant in the NMO-IgG+ dataset (rs1150757) after **conditioning on rs28383224.** The *x*-axis represents the chromosomal position; the *y*-axis represents the –Log_{10}P-value of association. Colors indicate the linkage disequilibrium (LD) level with the index SNP. Purple lines represent recombination hotspots.

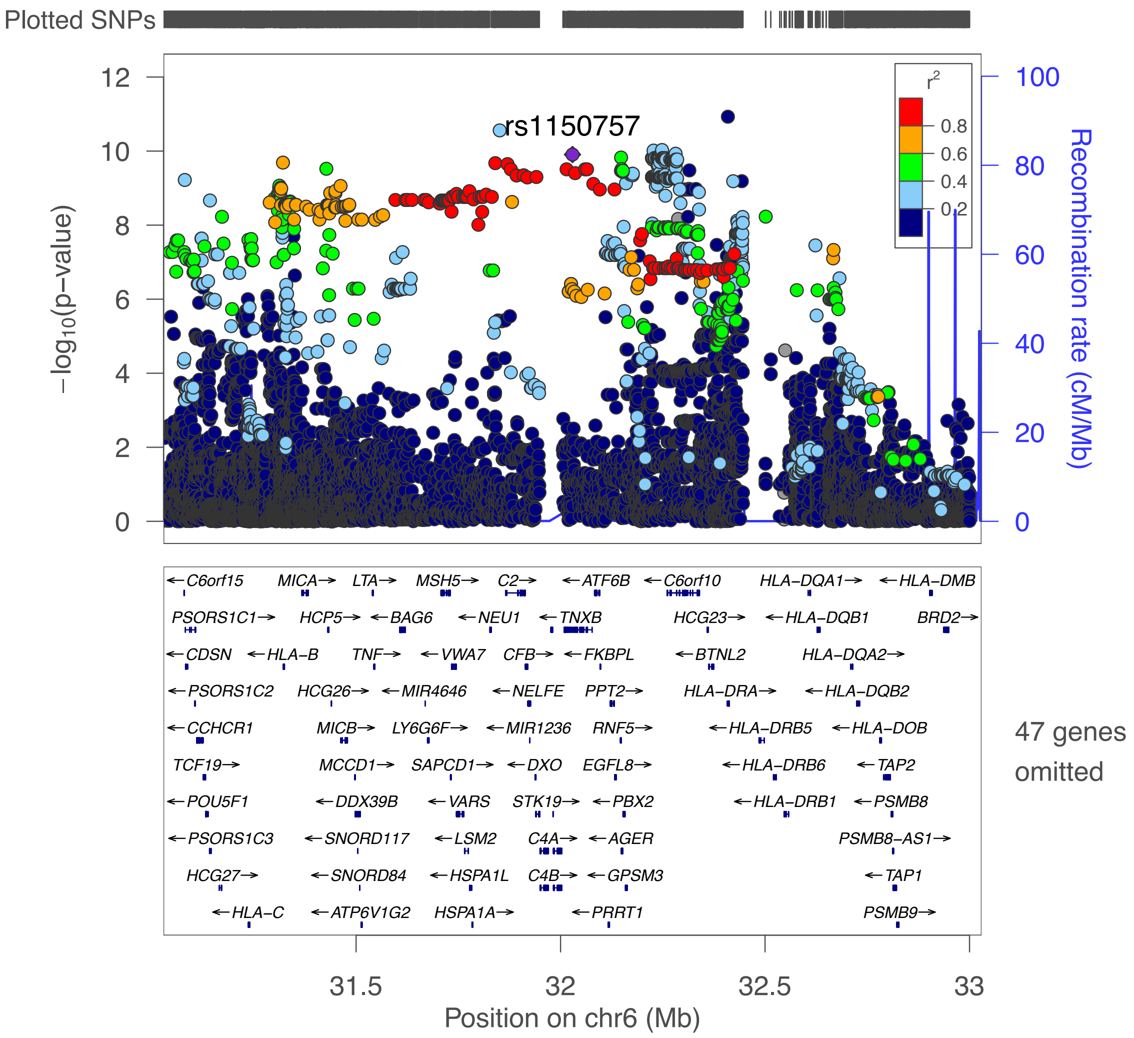

Supplementary Figure 6. Regional association plot of the most significant region in the NMO-IgG+ dataset after conditioning on HLA-DRB1*03:01. The *x*-axis represents the chromosomal position; the *y*-axis represents the $-\text{Log}_{10}$P-value of association. Colors indicate the linkage disequilibrium (LD) level with the index SNP. Purple lines represent recombination hotspots.

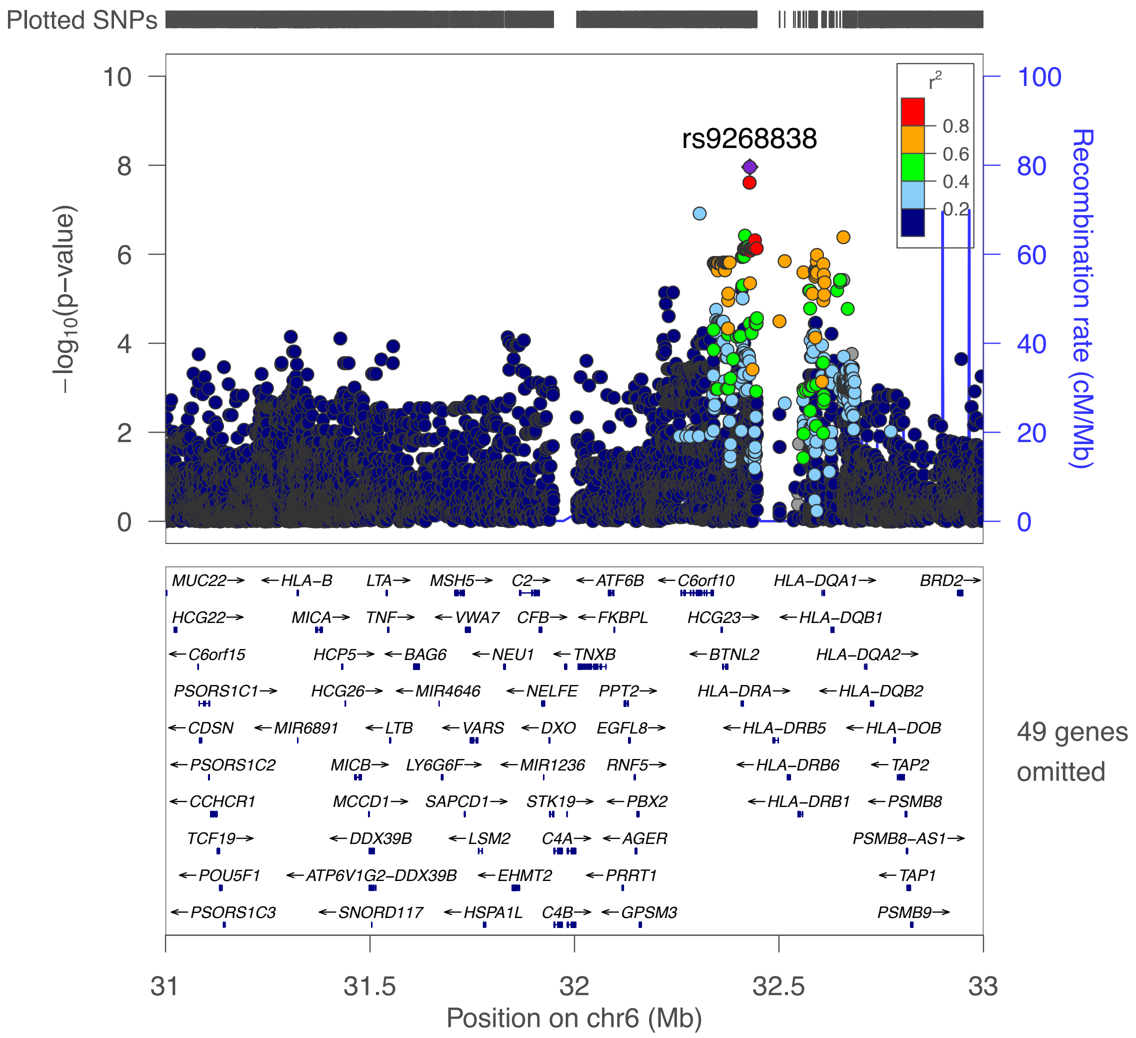

Supplementary Figure 7. Forest plot illustrating the effect of C4A and C4B copy number in NMO-IgG+.[1-3] Odds ratios represented by gray squares, where the size of the box is proportional to the inverse of their standard errors. Black lines represent the 95% confidence intervals of the odds ratios. Association results for each of the six most common haplotypes in the C4 locus are shown. **A.** Stage I. **B.** Stage II.

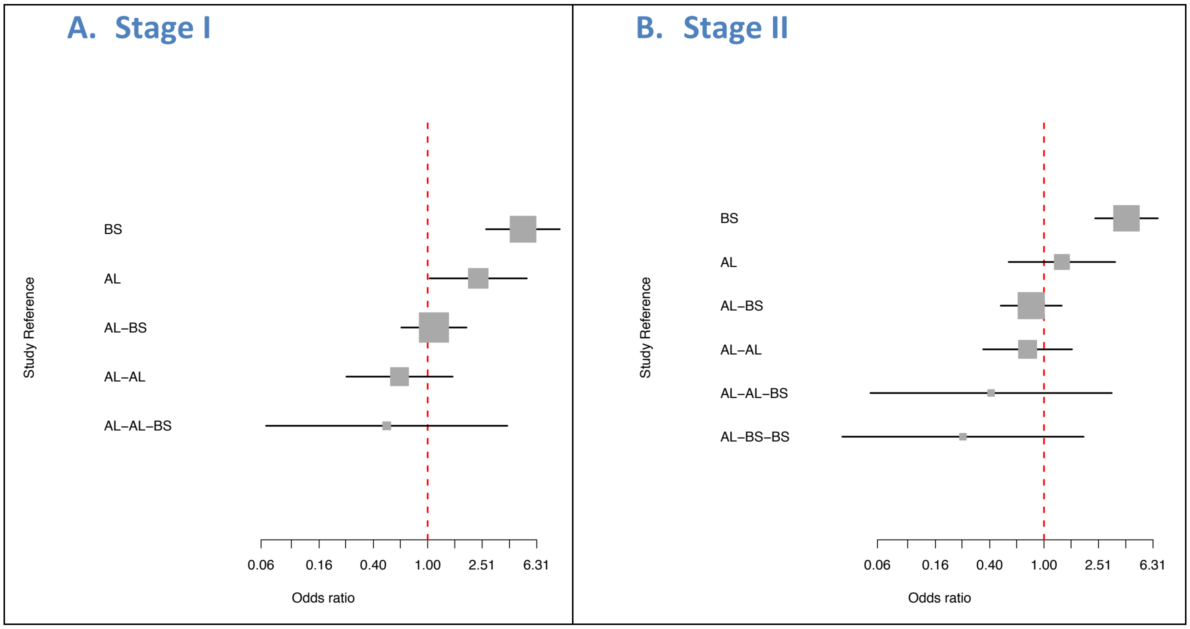

[1] BS, haplotype carrying only the short form of C4B;
AL, haplotype carrying only the long form of C4A;
AL-BS, haplotype carrying one copy of the long form of C4A and the short form of C4B;
AL-AL, haplotype carrying two copies of the long form of C4A;
AL-AL-BS, haplotype carrying two copies of the long form of C4A and one of the short form of C4B; and
AL-BS-BS, haplotype carrying one copy of the long form of C4A and two copies of the short form of C4B.

[2] The most common haplotype (one copy of the long forms of C4A and C4B) was used as a reference (OR=1) for comparison to the other haplotypes.

[3] The AL-BS-BS haplotype is not depicted for Stage I because it was too rare to provide stable statistics.

Supplementary Figure 8. Forest plot showing the effect of C4A and C4B copy number in the NMO-IgG+ dataset. Effects are regardless of C4 size: short or long. The most common number of total C4A and C4B (2) was used as a reference (OR=1).

C4 type and number	OR	P
C4A_0	5.26	0.008
C4A_1	5.13	4.85E-14
C4A_3	0.30	0.002
C4A_4	0.23	0.195
C4B_0	1.96	0.552
C4B_1	2.38	0.001
C4B_3	0.51	0.191

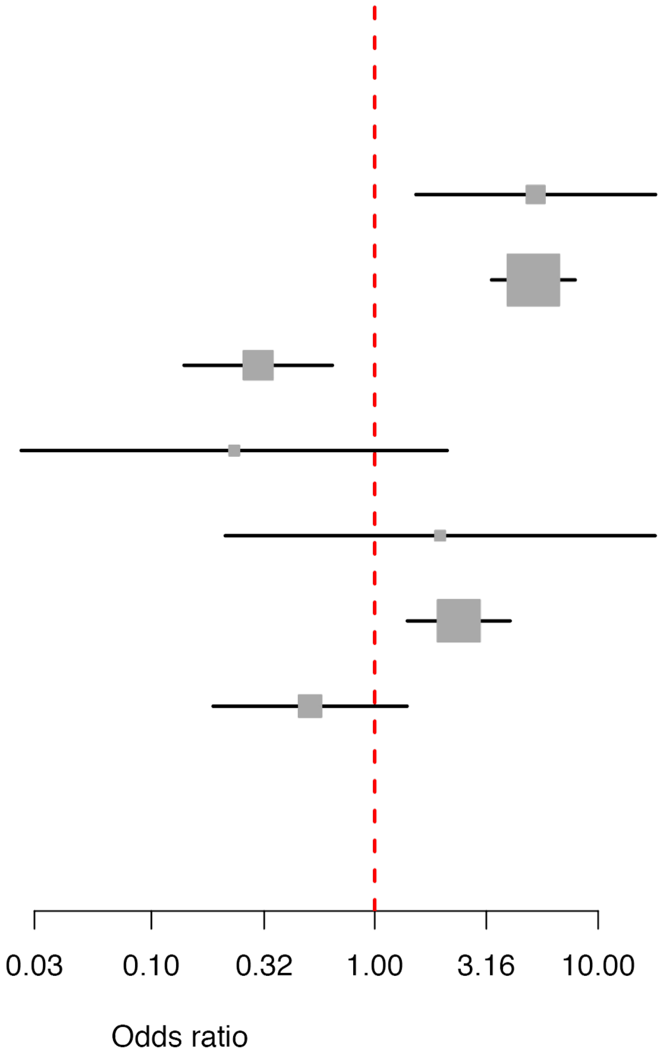

Supplementary Figure 9. Effect of rs1150757 on C4A expression. The genetic variant rs1150757 was significantly associated with various tissues in the GTEX project.

Multi-tissue eQTL Comparison

ENSG00000244731.3 C4A and rs1150757 eQTL (Meta Analysis RE2 P-Value: 0)

Tissue	Samples	Beta	p-value	post-prob
Uterus	70	-	-	-
Nerve - Tibial	256	-1.23	5.5e-23	1.00
Thyroid	278	-0.914	1.2e-20	1.00
Esophagus - Muscularis	218	-0.885	1.1e-19	1.00
Muscle - Skeletal	361	-0.969	2.0e-19	1.00
Adipose - Subcutaneous	298	-0.975	3.0e-18	1.00
Skin - Sun Exposed (Lower leg)	302	-1.10	6.2e-18	1.00
Stomach	170	-1.03	1.1e-17	1.00
Cells - Transformed fibroblasts	272	-0.649	1.2e-17	1.00
Esophagus - Mucosa	241	-1.15	1.3e-17	1.00
Lung	278	-0.833	1.9e-17	1.00
Testis	157	-1.41	1.3e-16	1.00
Whole Blood	338	-0.934	2.3e-16	1.00
Skin - Not Sun Exposed (Suprapubic)	196	-1.27	7.5e-16	1.00
Artery - Aorta	197	-0.940	8.9e-16	1.00
Heart - Left Ventricle	190	-0.804	1.2e-15	1.00
Heart - Atrial Appendage	159	-0.912	2.0e-15	1.00
Liver	97	-0.885	7.0e-14	1.00
Artery - Tibial	285	-0.944	8.9e-14	1.00
Pancreas	149	-0.817	1.3e-13	1.00
Breast - Mammary Tissue	183	-0.888	1.3e-13	1.00
Colon - Transverse	169	-0.826	2.2e-13	1.00
Esophagus - Gastroesophageal Junction	127	-0.920	5.9e-11	1.00
Adipose - Visceral (Omentum)	185	-0.607	6.0e-11	1.00
Cells - EBV-transformed lymphocytes	114	-1.29	8.4e-10	1.00
Brain - Frontal Cortex (BA9)	92	-0.869	5.6e-9	1.00
Spleen	89	-1.36	6.2e-9	1.00
Adrenal Gland	126	-0.857	1.2e-8	1.00
Colon - Sigmoid	124	-0.904	1.4e-8	1.00
Brain - Cerebellum	103	-1.30	3.0e-8	1.00
Brain - Cerebellar Hemisphere	89	-1.50	6.1e-8	1.00
Prostate	87	-1.00	6.5e-7	1.00
Brain - Hypothalamus	81	-0.832	1.0e-6	1.00
Artery - Coronary	118	-0.922	1.9e-6	1.00
Brain - Anterior cingulate cortex (BA24)	72	-0.966	2.4e-6	1.00
Brain - Putamen (basal ganglia)	82	-0.964	2.8e-6	1.00
Brain - Caudate (basal ganglia)	100	-0.760	4.6e-6	1.00
Pituitary	87	-1.01	5.4e-6	1.00
Ovary	85	-0.973	1.3e-5	1.00
Vagina	79	-0.841	5.3e-5	1.00
Small Intestine - Terminal Ileum	77	-0.628	1.8e-4	1.00
Brain - Hippocampus	81	-0.646	5.1e-4	1.00
Brain - Cortex	96	-0.609	2.5e-3	1.00
Brain - Nucleus accumbens (basal ganglia)	93	-0.575	0.01	1.00

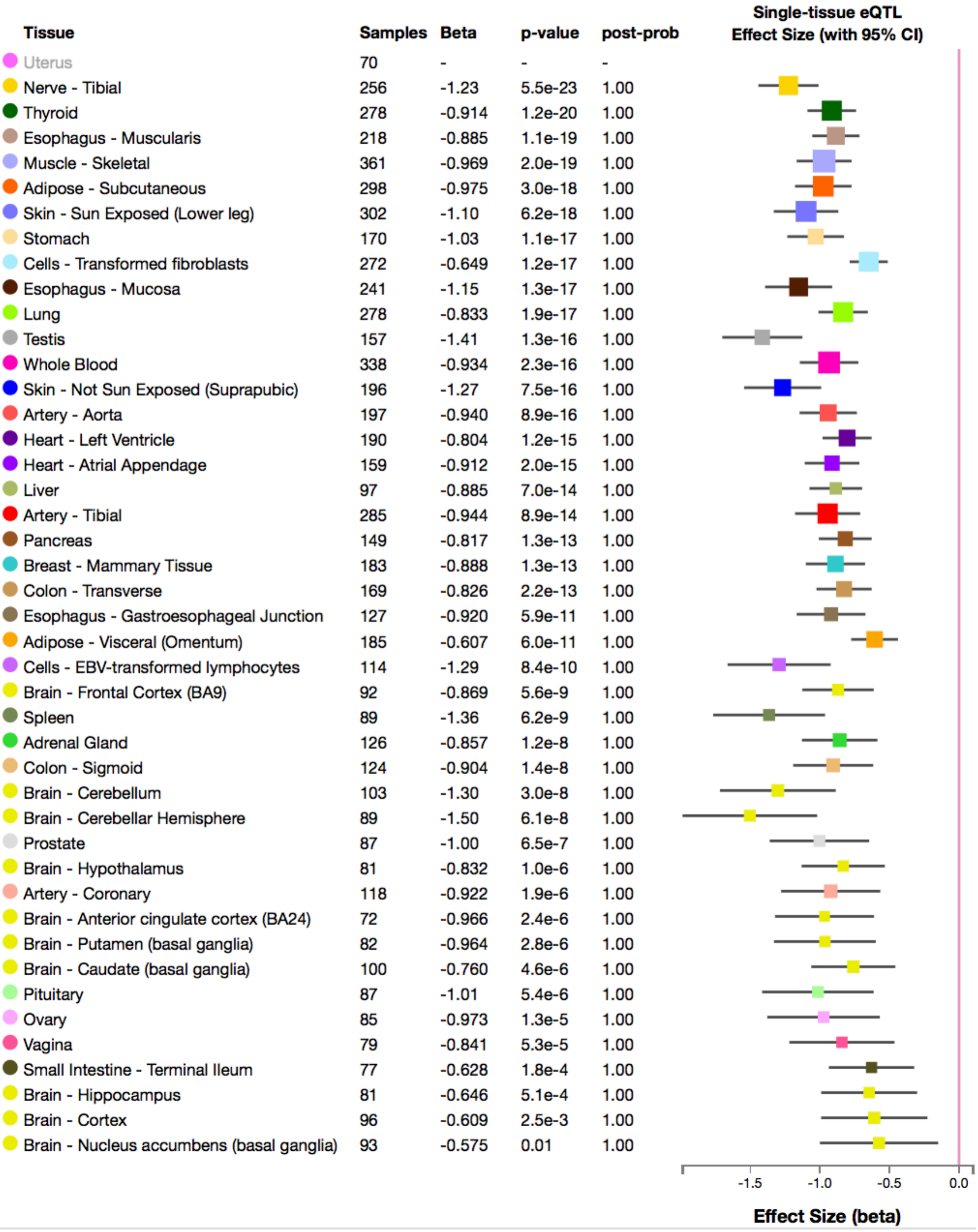

Supplementary Figure 10. Gene expression profile of C4A in nerve tibial tissue. The rank normalized expression of C4A is depicted in the different genotype classes of the snp rs1150757. Results for nerve tibial tissue from the GTEX project.

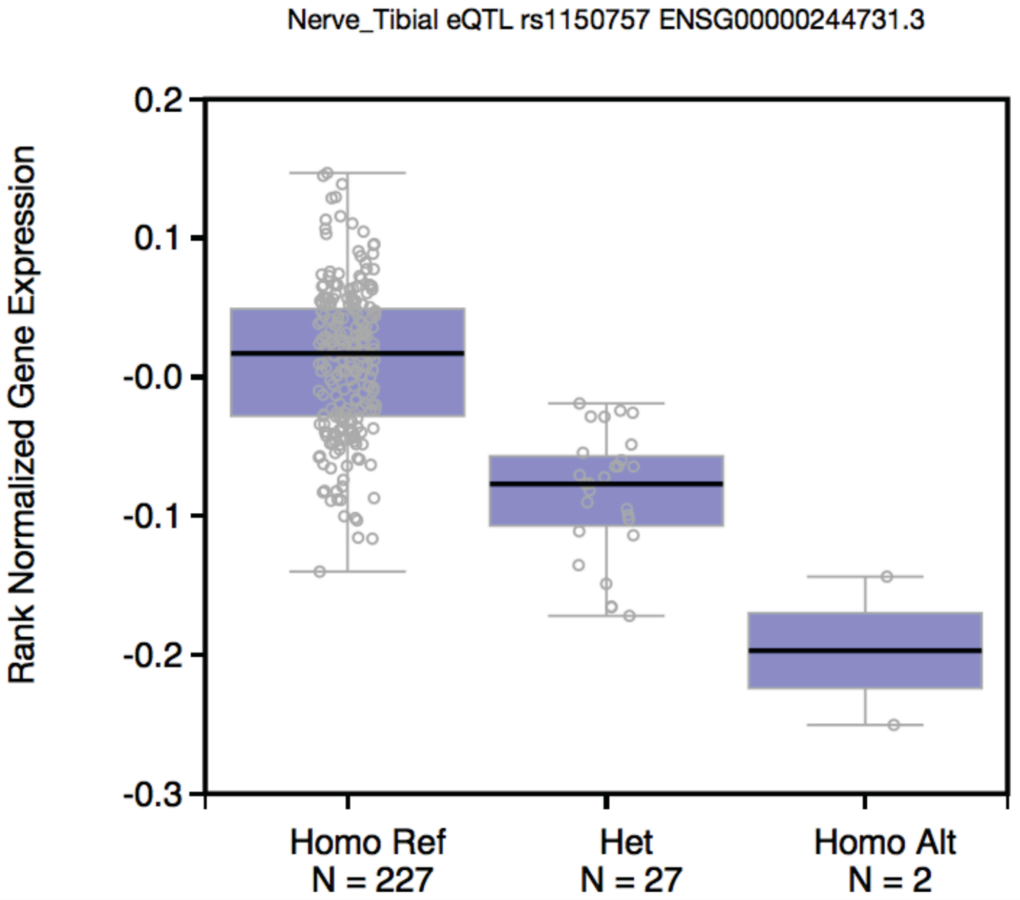

Homo Ref, GG; Het GA; Homo Alt AA

Supplementary Figure 11. Mendelian randomization (MR) leave one out analysis of previously identified SLE risk variants in IgG-NMO+. Each line represents the MR effect of the SLE variants after excluding the genetic marker depicted on the left.

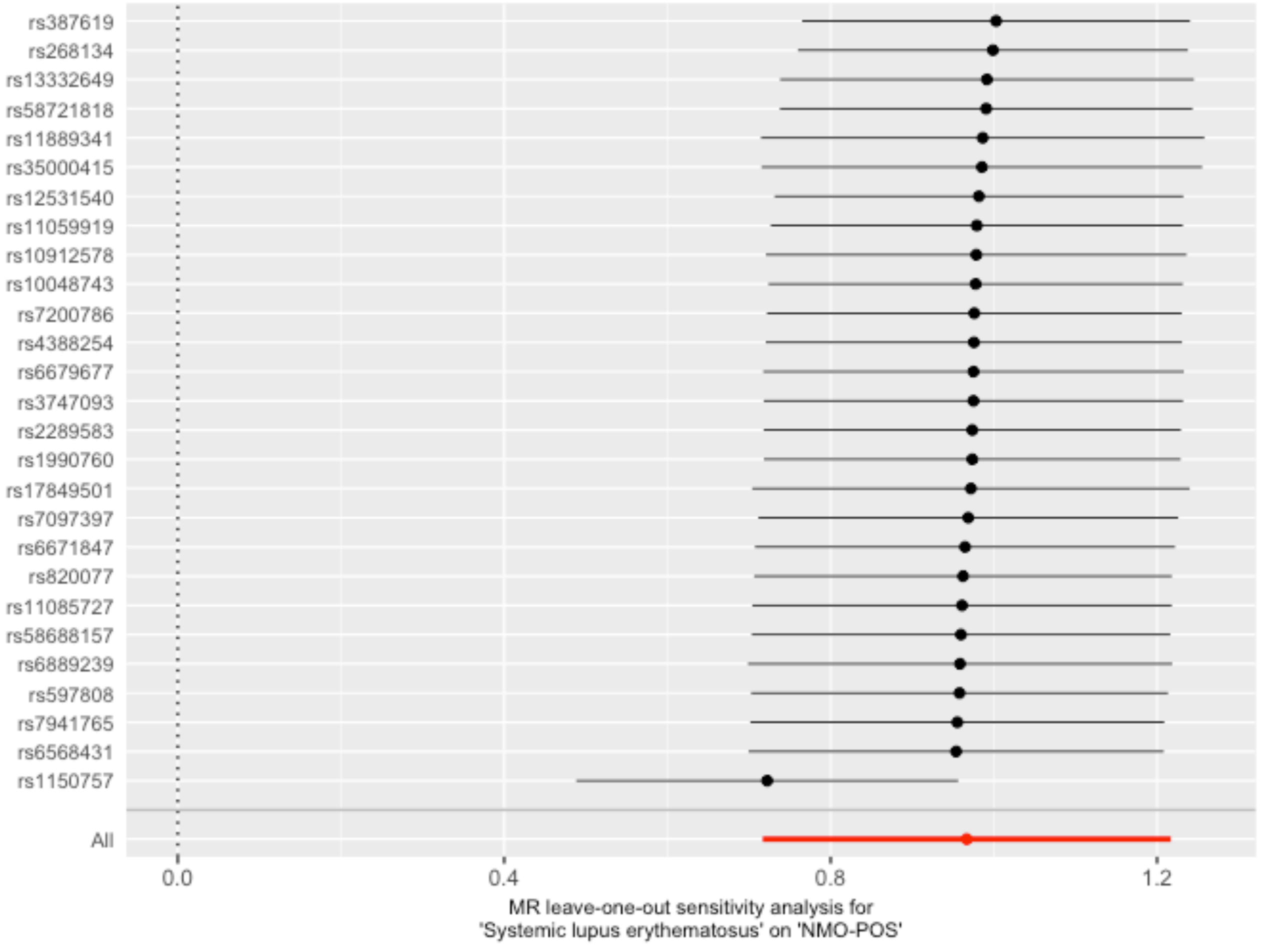

Supplement: Supplementary file 1 — Supplementary Information [file 41467_2018_4332_MOESM1_ESM.pdf]
